# Supplementary material for: A retrospective study on expression and clinical significance of PHH3, Ki67 and P53 in bladder exophytic papillary urothelial neoplasms
Source: PeerJ. 2023 Jul 11;11:e15675. doi: 10.7717/peerj.15675 (PMC10348311; doi:10.7717/peerj.15675)
Supplement: Supplemental Information 4 [file peerj-11-15675-s004.docx]

CROSSTABS

/TABLES= Gender

Age were grouped BY 65 years old

History of drinking

History of smoking

Tumor volume

Whether recurrence

Whether death

Depth of invasion

Clinical stage

By PHH3 13.5 as the boundary

By HE 14.5 as the boundary. By he 14.5 as the boundary

By KI67 9.5 percent grouping

By P53 50 percent grouping

/FORMAT=AVALUE TABLES

/STATISTICS=CHISQ CC PHI

/CELLS=COUNT ROW COLUMN TOTAL

/COUNT ROUND CELL

/BARCHART

/METHOD=EXACT TIMER (5).

**crosstabs**

**Remarks**

| Output created | | 22-MAY-2023 16:39:34  C:\Users\ Qi Gaoxiu \Desktop\PeerJ contribution \ urothelium ②.sav  Data Set 1  < None >  < None >  < None >  101 |
| --- | --- | --- |
| Notes  Input | Data  Activity data set  Filters  weight  Split file  Number of lines in the working data file |  |

| Missing Value Processing | Definition of missing value  Number of cases used | Treat a user-defined missing value as missing.  Statistics for each table are based on all cases in each table that meet the following conditions: For all variables, there is valid data within the specified range. |
| --- | --- | --- |
| Syntax |  | CROSSTABS  /TABLES= Gender age group 65 years old drinking history smoking history tumor volume recurrence death depth of infiltration Clinical stage BY PHH3 limit 13.5 HE 14.5 KI67 group 9.5 percent  P53 was bounded by 50 percent /FORMAT=AVALUE TABLES /STATISTICS=CHISQ CC PHI /CELLS=COUNT ROW COLUMN  TOTAL  /COUNT ROUND CELL /BARCHART /METHOD=EXACT TIMER(5). |
| Resources | Processor time Use up time Requested dimension Available cells  Time for precise  statistics | 00:00:03. 56 |
|  |  | 00:00:03. 17 |
|  |  | 2 |
|  |  | 524245 |
|  |  | 0:00:00. 09 |

**Case** **summary**

|  | Case | | | | | |
| --- | --- | --- | --- | --- | --- | --- |
|  | Valid | | Missing | | Total | |
|  | N | Percentag e | N | Percentag e | N | Percentag e |
| Gender * PHH3 is bounded by 13.5  Gender * HE is bounded by 14.5  Gender * KI67 grouped by 9.5 percent | 101  101  101 | 100.0%  100.0%  100.0% | 0  0  0 | 0.0%  0.0%  0.0% | 101  101  101 | 100.0%  100.0%  100.0% |

100.0%

100.0%

100.0%

100.0%

100.0%

100.0%

100.0%

100.0%

100.0%

100.0%

100.0%

100.0%

100.0%

100.0%

100.0%

100.0%

100.0%

100.0%

100.0%

100.0%

Gender * P53 is limited by 50 percent

Age is grouped by 65 years * PHH3 with a boundary of 13.5

Age is grouped by 65 years * HE is bounded by 14.5 Age grouped by 65 years * KI67 by 9.5 percent Age group by 65 years * P53 is bounded by 50 percent

Alcohol history * PHH3 is bounded by 13.5

Alcohol history * HE has a boundary of 14.5

Alcohol history * KI67 grouped by 9.5 percent Alcohol history * P53 is delimited by 50 percent Smoking history * PHH3 is bounded by 13.5 Smoking history * HE has a boundary of 14.5 Smoking history * KI67 grouped by 9.5 percent Smoking history * P53 is bounded by 50 percent Volume of mass * PHH3 with a limit of 13.5 Volume of mass * HE is bounded by 14.5

Volume of mass * KI67 grouped by 9.5 percent Tumor volume * P53 is limited by 50 percent Relapse or not * PHH3 with a limit of 13.5

Relapse or not * HE is limited by 14.5 Recurrence or not * KI67 grouped by 9.5 percent

101

101

101

101

101

101

101

101

101

101

101

101

101

101

101

101

101

101

101

101

| 0  0  0  0  0  0  0  0  0  0  0  0  0  0  0  0  0  0  0  0 | \| 101  101  101  101  101  101  101  101  101  101  101  101  101  101  101  101  101  101  101  101 \| 100.0%  100.0%  100.0%  100.0%  100.0%  100.0%  100.0%  100.0%  100.0%  100.0%  100.0%  100.0%  100.0%  100.0%  100.0%  100.0%  100.0%  100.0%  100.0%  100.0% \| \| --- \| --- \|   0.0%  0.0%  0.0%  0.0%  0.0%  0.0%  0.0%  0.0%  0.0%  0.0%  0.0%  0.0%  0.0%  0.0%  0.0%  0.0%  0.0%  0.0%  0.0%  0.0% |
| --- | --- | --- | --- |

| Relapse or not * P53 is bounded by 50 percent Dead or not * PHH3 with a boundary of 13.5  Dead or not * HE is  bounded by 14.5  Dead or not * KI67 grouped by 9.5 percent Dead or not * P53 is bounded by 50 percent Depth of infiltration * PHH3 with a limit of 13.5 Depth of infiltration * HE is limited by 14.5 Infiltration depth * KI67 grouped in 9.5 percent Infiltration depth * P53 with 50 percent boundary Clinical staging * PHH3 with a limit of 13.5 Clinical staging * HE was limited by 14.5  Clinical staging * KI67 grouped by 9.5 percent Clinical stage * 50 percent P53 boundary | 101  101  101  101  101  101  101  101  101  101  101  101  101 | 100.0%  100.0%  100.0%  100.0%  100.0%  100.0%  100.0%  100.0%  100.0%  100.0%  100.0%  100.0%  100.0% | 0  0  0  0  0  0  0  0  0  0  0  0  0 | 0.0%  0.0%  0.0%  0.0%  0.0%  0.0%  0.0%  0.0%  0.0%  0.0%  0.0%  0.0%  0.0% | 101  101  101  101  101  101  101  101  101  101  101  101  101 | 100.0%  100.0%  100.0%  100.0%  100.0%  100.0%  100.0%  100.0%  100.0%  100.0%  100.0%  100.0%  100.0% |
| --- | --- | --- | --- | --- | --- | --- |

**Gender** ***** **PHH3** **is** **bounded** **by** **13.5**

**crosstabs**

|  | | | PHH3 is bounded by 13.5 | | Total |
| --- | --- | --- | --- | --- | --- |
|  |  |  | 13 or less | Greater than  or equal to  14 |  |
| Gende r | male | Counting  Percentage of gender | 40 | 38 | 78 |
|  |  |  | 51.3% | 48.7% | 100.0% |

| Percentage of PHH3 bounded by 13.5  Percentage of total  female count   \| As a percentage of gender \| \| --- \| \| Percentage of PHH3  bounded by 13.5 \|   Percentage of total | | 75. 5% | 79.2% | 77.2% |
| --- | --- | --- | --- | --- | --- | --- |
|  |  | 39.6% | 37.6% | 77.2% |
|  |  | 13 | 10 | 23 |
|  |  | 56. 5% | 43. 5% | 100.0% |
|  |  | 24. 5% | 20.8% | 22.8% |
|  |  | 12.9% | 9.9% | 22.8% |
| total | Counting   \| Percentage of gender \| \| --- \| \| Percentage of PHH3  bounded by 13.5 \|   Percentage of total | 53 | 48 | 101 |
|  |  | 52. 5% | 47. 5% | 100.0% |
|  |  | 100.0% | 100.0% | 100.0% |
|  |  | 52. 5% | 47. 5% | 100.0% |

**Chi-square** **test**

|  | value | Degrees  of  freedom | Progressive  significance  (bilateral) | Precise  significance  (bilateral) | Exact  significance  (unilateral) | Point  probabilit  y |
| --- | --- | --- | --- | --- | --- | --- |
| Pearson chi- square Continuity correction Likelihood ratio Fisher precision test  Linear correlation Number of valid cases | 196.  042. 196.  194.  101 | 1  1  1  1 | 658.  838. 658.  660. | 813.  813. 813.  813. | 420.  420. 420.  420. | 171. |

a. 0 cells (0.0%) have an expected count of less than 5. The minimum expected count is 10.93.

b. Calculate for 2x2 tables only

c. Standardized statistics are -.440.

**Symmetrical** **measurement**

|  | value | Progressive significance | Precise significance |
| --- | --- | --- | --- |
| Nominal to Phi | - 044. | 658. | c  . |

| nominal Klem V  Number of column connectio ns  Number of valid cases | 044. | 658. | c  . |
| --- | --- | --- | --- |
|  | 044.  101 | 658. | c  . |

c. Calculations cannot be performed because the temporary file

cannot be opened.

**Gender** ***** **HE** **is** **bounded** **by** **14.5**

**crosstabs**

|  | | | HE is bounded by 14.5 | | Total |
| --- | --- | --- | --- | --- | --- |
|  |  |  | 14 or less | 15 or  more |  |
| Gende male Counting | | | 42 | 36 | 78 |
| r | Percentage of gender  Percentage of HE bounded by 14.5  Percentage of total  female Counting   \| As a percentage of gender \| \| --- \| \| Percentage of HE bounded by 14.5 \| | |  |  |  |
|  |  |  | 53.8% | 46.2% | 100.0% |
|  |  |  | 75.0% | 80.0% | 77.2% |
|  |  |  | 41.6% | 35.6% | 77.2% |
|  |  |  | 14 | 9 | 23 |
|  |  |  | 60.9% | 39. 1% | 100.0% |
|  |  |  | 25.0% | 20.0% | 22.8% |
|  |  |  | 13.9% | 8.9% | 22.8% |
| A percentage of the total | | |  |  |  |
| Total | | Counting   \| Percentage of gender \| \| --- \| \| Percentage of HE bounded by 14.5 \|   Percentage of total | 56 | 45 | 101 |
|  |  |  | 55.4% | 44.6% | 100.0% |
|  |  |  | 100.0% | 100.0% | 100.0% |
|  |  |  | 55.4% | 44.6% | 100.0% |

**Chi-square** **test**

|  | value | Degrees  of  freedom | Progressive  significance  (bilateral) | Precise  significance  (bilateral) | Exact  significance  (unilateral) | Point  probabilit  y |
| --- | --- | --- | --- | --- | --- | --- |
| Pearson chi- square Continuity correction Likelihood ratio Fisher precision test  Linear correlation Number of valid cases | 355.  127. 357.  351.  101 | 1  1  1  1 | 551.  721. 550.  553. | 637.  637. 637.  637. | 362.  362. 362.  362. | 160. |

a. 0 cells (0.0%) have an expected count of less than 5. The minimum expected count is 10.25.

b. Calculate for 2x2 tables only

c. Standardized statistics are -.593.

**Symmetrical** **measurement**

|  | | value | Progressive significance | Precise significance |
| --- | --- | --- | --- | --- |
| Nominal to Phi | | - 059. | 551. | c  . |
| nominal | Clem V |  |  |  |
|  |  | 059. | 551. | c  . |
|  |  | 059.  101 | 551. | c  . |
| Number of column connectio ns  Number of valid cases | |  |  |  |

c. Calculations cannot be performed because the temporary file

cannot be opened.

**Gender** ***** **KI67** **grouped** **by** **9.5** **percent**

**crosstab**

|  | | | | KI67 is grouped by 9.5 percent | | Total |
| --- | --- | --- | --- | --- | --- | --- |
|  |  |  |  | 9 or less | 10 or  higher |  |
| Gende | | male | Counting | 32 | 46 | 78 |
| r | Percentage of gender  Percentage of KI67 grouped by 9.5 percent  Percentage of total  female Counting   \| As a percentage of gender \| \| --- \| \| Accounts for 9.5 percent of KI67 groups \|   Percentage of total | | | 41.0% | 59.0% | 100.0% |
|  |  |  |  | 74.4% | 79.3% | 77.2% |
|  |  |  |  | 31.7% | 45. 5% | 77.2% |
|  |  |  |  | 11 | 12 | 23 |
|  |  |  |  | 47.8% | 52.2% | 100.0% |
|  |  |  |  | 25.6% | 20.7% | 22.8% |
|  |  |  |  | 10.9% | 11.9% | 22.8% |
| Total | | Counting   \| Percentage of gender \| \| --- \| \| Percentage of KI67 grouped by 9.5 percent \|   Percentage of total | | 43 | 58 | 101 |
|  |  |  |  | 42.6% | 57.4% | 100.0% |
|  |  |  |  | 100.0% | 100.0% | 100.0% |
|  |  |  |  | 42.6% | 57.4% | 100.0% |

**Chi-square** **test**

|  | value | Degrees  of  freedom | Progressive  significance  (bilateral) | Precise  significance  (bilateral) | Exact  significance  (unilateral) | Point  probabilit  y |
| --- | --- | --- | --- | --- | --- | --- |
| Pearson chi- square Continuity correction Likelihood ratio Fisher's exact test | 336.  115. 334. | 1  1  1 | 562.  734. 563. | 634.  634.  634. | 365.  365.  365. |  |

a. 0 cells (0.0%) have an expected count of less than 5. The minimum expected count is 9.79.

b. Calculate for 2x2 tables only

c. Standardized statistics are -.577.

**Symmetrical** **measurement**

|  | | value | Progressive significance | Precise significance |
| --- | --- | --- | --- | --- |
| Nominal to Phi | | - 058. | 562. | c  . |
| nominal | Clem V |  |  |  |
|  |  | 058. | 562. | c  . |
|  |  | 058.  101 | 562. | c  . |
| Number of column connectio ns  Number of valid cases | |  |  |  |

c. Calculations cannot be performed because the temporary file

cannot be opened

**Gender** ***** **P53** **is** **bounded** **by** **50** **percent**

**crosstabs**

|  | | | P53 is bounded by 50 percent | | Total |
| --- | --- | --- | --- | --- | --- |
|  |  |  | < 50% | 50% or  higher |  |
| Gende | male | Counting | 60 | 18 | 78 |
| r | Percentage of gender  Percentage of P53 bounded by 50 percent  Percentage of total | | 76.9% | 23. 1% | 100.0% |
|  |  |  | 75.9% | 81.8% | 77.2% |
|  |  |  | 59.4% | 17.8% | 77.2% |

| female Counting   \| As a percentage of sex \| \| --- \| \| Percentage of P53 bounded by 50 percent \|   Percentage of total | | 19 | 4 | 23 |
| --- | --- | --- | --- | --- | --- | --- |
|  |  | 82.6% | 17.4% | 100.0% |
|  |  | 24. 1% | 18.2% | 22.8% |
|  |  | 18.8% | 4.0% | 22.8% |
| Total | Counting   \| Percentage of gender \| \| --- \| \| Percentage of P53 bounded by 50 percent \|   Percentage of total | 79 | 22 | 101 |
|  |  | 78.2% | 21.8% | 100.0% |
|  |  | 100.0% | 100.0% | 100.0% |
|  |  | 78.2% | 21.8% | 100.0% |

**Chi-square** **test**

|  | value | Degrees  of  freedom | Progressive  significance  (bilateral) | Precise  significance  (bilateral) | Exact  significance  (unilateral) | Point  probabilit  y |
| --- | --- | --- | --- | --- | --- | --- |
| Pearson chi- square Continuity correction Likelihood ratio Fisher precision test  Linear correlation Number of valid cases | 337.  086. 350.  334.  101 | 1  1  1  1 | 562.  769. 554.  564. | 590.  590. 775.  590. | 396.  396. 396.  396. | 201. |

a. 0 cells (0.0%) have an expected count of less than 5. The minimum expected count is 5.01.

b. Calculate for 2x2 tables only

c. Standardized statistics are -.578.

**Symmetrical** **measurement**

|  | value | Progressive significance | Precise significance |
| --- | --- | --- | --- |
| Nominal to Phi  nominal Clem V | - 058. | 562. | c  . |
|  | 058. | 562. | c  . |

| Number of column connectio ns  Number of valid cases | 058.  101 | 562. | c  . |
| --- | --- | --- | --- |

c. Calculations cannot be performed because the temporary file

cannot be opened.

**Age** **is** **grouped** **by** **65** **years** ***** **PHH3** **is** **bounded** **by** **13.5**

|  | | PHH3 is bounded by 13.5 | | Total |
| --- | --- | --- | --- | --- |
|  |  | Less than or equal to 13 | Greater than  or equal to  14 |  |
| Age groups are 65 years old | < 65 Count  Percentage of age grouped by 65 years  Percentage of PHH3  bounded by 13.5  A percentage of the total  P 65 Count   \| Percentage of age grouped by 65 \| \| --- \| \| Percentage of PHH3  bounded by 13.5 \|   Percentage of total | 30 | 14 | 44 |
|  |  | 68.2% | 31.8% | 100.0% |
|  |  | 56.6% | 29.2% | 43.6% |
|  |  | 29.7% | 13.9% | 43.6% |
|  |  | 23 | 34 | 57 |
|  |  | 40.4% | 59.6% | 100.0% |
|  |  | 43.4% | 70.8% | 56.4% |
|  |  | 22.8% | 33.7% | 56.4% |
| Total | Counting   \| Accounting for the  percentage of age  grouped by 65 \| \| --- \| \| Percentage of PHH3  bounded by 13.5 \|   Percentage of total | 53 | 48 | 101 |
|  |  | 52. 5% | 47. 5% | 100.0% |
|  |  | 100.0% | 100.0% | 100.0% |
|  |  | 52. 5% | 47. 5% | 100.0% |

**Chi-square** **test**

|  | value | Degrees  of  freedom | Progressive  significance  (bilateral) | Precise  significance  (bilateral) | Exact  significance  (unilateral) | Point  probabilit  y |
| --- | --- | --- | --- | --- | --- | --- |
| Pearson chi- square Continuity correction Likelihood ratio Fisher precision test  Linear correlation Number of valid cases | 7.712a  6.637 7.842  7.636c  101 | 1  1  1  1 | 005.  010. 005.  006. | 009.  009. 009.  009. | 005.  005. 005.  005. | 003. |

a. 0 cells (0.0%) have an expected count of less than 5. The minimum expected count is 20.91.

b. Calculate for 2x2 tables only

c. 2.763 for standardized statistics.

**Symmetrical** **measurement**

|  | | value | Progressive significance | Precise significance |
| --- | --- | --- | --- | --- |
| Nominal to Phi | | 276. | 005. | c  . |
| nominal | Clem V |  |  |  |
|  |  | 276. | 005. | c  . |
|  |  | 266.  101 | 005. | c  . |
| Number of column connectio ns  Number of valid cases | |  |  |  |

c. Calculations cannot be performed because the temporary file

cannot be opened.

**Age** **group** **is** **65** **years** **old** ***** **HE** **is** **limited** **by** **14.5**

**Crosstabs**

|  | | HE is bounded by 14.5 | | Total |
| --- | --- | --- | --- | --- |
|  |  | 14 or less | 15 or  more |  |
| Age groups are 65 years old | < 65 Count  Percentage of age grouped by 65 years  Accounts for the percentage of HE bounded by 14.5  Percentage of total  P 65 Count   \| Percentage of age grouped by 65 \| \| --- \| \| Percentage of HE bounded by 14.5 \|   Percentage of total | 31 | 13 | 44 |
|  |  | 70. 5% | 29. 5% | 100.0% |
|  |  | 55.4% | 28.9% | 43.6% |
|  |  | 30.7% | 12.9% | 43.6% |
|  |  | 25 | 32 | 57 |
|  |  | 43.9% | 56. 1% | 100.0% |
|  |  | 44.6% | 71. 1% | 56.4% |
|  |  | 24.8% | 31.7% | 56.4% |
| Total | Counting   \| Accounting for the  percentage of age  grouped by 65 \| \| --- \| \| Percentage of HE bounded by 14.5 \|   Percentage of total | 56 | 45 | 101 |
|  |  | 55.4% | 44.6% | 100.0% |
|  |  | 100.0% | 100.0% | 100.0% |
|  |  | 55.4% | 44.6% | 100.0% |

**Chi-square** **test**

|  | value | Degrees  of  freedom | Progressive  significance  (bilateral) | Precise  significance  (bilateral) | Exact  significance  (unilateral) | Point  probabilit  y |
| --- | --- | --- | --- | --- | --- | --- |

| Pearson chi- square Continuity correction Likelihood ratio Fisher precision test  Linear correlation Number of valid cases | 7.110a  6.074 7.246  7.039c  101 | 1  1  1  1 | 008.  014. 007.  008. | 009.  009. 009.  009. | 007.  007. 007.  007. | 005. |
| --- | --- | --- | --- | --- | --- | --- |

a. 0 cells (0.0%) have an expected count of less than 5. The minimum expected count is 19.60.

b. Calculate for 2x2 tables only

c. 2.653 for standardized statistics.

**Symmetry** **measurement**

|  | | value | Progressive significance | Precise significance |
| --- | --- | --- | --- | --- |
| Nominal to Phi | | 265. | 008. | c  . |
| nominal | Clem V |  |  |  |
|  |  | 265. | 008. | c  . |
|  |  | 256.  101 | 008. | c  . |
| Number of column connectio ns  Number of valid cases | |  |  |  |

c. Calculations cannot be performed because the temporary file

cannot be opened

**Age** **is** **grouped** **by** **65** ***** **KI67** **by** **9.5** **percent**

**crosstabs**

|  | | | | KI67 is grouped by 9.5 percent | | Total |
| --- | --- | --- | --- | --- | --- | --- |
|  |  |  |  | 9 or less | 10 or  higher |  |
| Age groups are 65 years old | < | 65 | Count  Percentage of age grouped by 65 years  Percentage of KI67 grouped by 9.5 percent | 25 | 19 | 44 |
|  |  |  |  | 56.8% | 43.2% | 100.0% |
|  |  |  |  | 58. 1% | 32.8% | 43.6% |

| Percentage of total  P 65 Count   \| Percentage of age grouped by 65 \| \| --- \| \| Percentage of KI67 grouped by 9.5 percent \|   Percentage of total | | 24.8% | 18.8% | 43.6% |
| --- | --- | --- | --- | --- | --- | --- |
|  |  | 18 | 39 | 57 |
|  |  | 31.6% | 68.4% | 100.0% |
|  |  | 41.9% | 67.2% | 56.4% |
|  |  | 17.8% | 38.6% | 56.4% |
| Total | Counting   \| Accounting for the  percentage of age  grouped by 65 \| \| --- \| \| Percentage of KI67 grouped by 9.5 percent \|   Percentage of total | 43 | 58 | 101 |
|  |  | 42.6% | 57.4% | 100.0% |
|  |  | 100.0% | 100.0% | 100.0% |
|  |  | 42.6% | 57.4% | 100.0% |

**Chi-square** **test**

|  | value | Degree of freedom | Progressive  significance  (bilateral) | Precise  significance  (bilateral) | Exact  significance  (unilateral) | Point  probabilit  y |
| --- | --- | --- | --- | --- | --- | --- |
| Pearson chi- square Continuity correction Likelihood ratio Fisher precision test  Linear correlation Number of valid cases | 6.470a  5.479 6.507  6.406c  101 | 1  1  1  1 | 011.  019. 011.  011. | 015.  015. 015.  015. | 010.  010. 010.  010. | 007. |

a. 0 cells (0.0%) have an expected count of less than 5. The minimum expected count is 18.73.

b. Calculate for 2x2 tables only

c. 2.531 for standardized statistics.

**Symmetrical** **measurement**

|  | value | Progressive significance | Precise significance |
| --- | --- | --- | --- |

| Nominal to Phi | | 253. | 011. | c  . |
| --- | --- | --- | --- | --- |
| nominal | Clem V |  |  |  |
|  |  | 253. | 011. | c  . |
|  |  | 245.  101 | 011. | c  . |
| Number of column connectio ns  Number of valid cases | |  |  |  |

c. Calculations cannot be performed because the temporary file

cannot be opened

**Age** **is** **grouped** **by** **65** **years** ***** **P53** **is** **bounded** **by** **50** **percent**

**Crosstabs**

|  | | P53 is bounded by 50 percent | | Total |
| --- | --- | --- | --- | --- |
|  |  | < 50% | 50% or  higher |  |
| Age group by 65 < 65 count | | 37 | 7 | 44 |
| years | Percentage of age grouped by 65 years  Percentage of P53 bounded by 50 percent  Percentage of total  P 65 Count   \| Percentage of age grouped by 65 \| \| --- \| \| Percentage of P53 bounded by 50 percent \| |  |  |  |
|  |  | 84. 1% | 15.9% | 100.0% |
|  |  | 46.8% | 31.8% | 43.6% |
|  |  | 36.6% | 6.9% | 43.6% |
|  |  | 42 | 15 | 57 |
|  |  | 73.7% | 26.3% | 100.0% |
|  |  | 53.2% | 68.2% | 56.4% |
|  |  | 41.6% | 14.9% | 56.4% |
| Percentage of total | |  |  |  |
| Total | Counting   \| Accounting for the  percentage of age  grouped by 65 \| \| --- \| \| Percentage of P53 bounded by 50 percent \|   Percentage of total | 79 | 22 | 101 |
|  |  | 78.2% | 21.8% | 100.0% |
|  |  | 100.0% | 100.0% | 100.0% |
|  |  | 78.2% | 21.8% | 100.0% |

**Chi-square** **test**

|  | value | Degrees  of  freedom | Progressive  significance  (bilateral) | Precise  significance  (bilateral) | Exact  significance  (unilateral) | Point  probabilit  y |
| --- | --- | --- | --- | --- | --- | --- |
| Pearson chi- square Continuity correction Likelihood ratio Fisher precision test  Linear  correlation | 1.578a  1.027 1.616  1.563c | 1  1  1  1 | 209.  311. 204.  211. | 234.  234. 234.  234. | 155.  155. 155.  155. | 090. |

a. 0 cells (0.0%) have an expected count of less than 5. The minimum expected count is 9.58.

b. Calculate for 2x2 tables only

c. 1.250 for standardized statistics.

**Symmetrical** **measurement**

|  | | value | Progressive significance | Precise significance |
| --- | --- | --- | --- | --- |
| Nominal to Phi | | 125. | 209. | c  . |
| nominal | Clem V |  |  |  |
|  |  | 125. | 209. | c  . |
|  |  | 124.  101 | 209. | c  . |
| Number of column connectio ns  Number of valid cases | |  |  |  |

c. Calculations cannot be performed because the temporary file

cannot be opened.

**Alcohol** **history** ***** **PHH3** **is** **bounded** **by** **13.5**

**Crosstabs**

|  | | | PHH3 is bounded by 13.5 | | Total |
| --- | --- | --- | --- | --- | --- |
|  |  |  | Less than or equal to 13 | Greater than  or equal to  14 |  |
| Drinkin g history | There is no | Count  Percentage of drinking history  Percentage of PHH3  bounded by 13.5 | 34 | 33 | 67 |
|  |  |  | 50.7% | 49.3% | 100.0% |
|  |  |  | 64.2% | 68.8% | 66.3% |

| A percentage of the total | | 33.7% | 32.7% | 66.3% |
| --- | --- | --- | --- | --- |
| There are | Counting   \| Percentage of drinking  history \| \| --- \| \| Percentage of PHH3  bounded by 13.5 \|   Percentage of total | 19 | 15 | 34 |
|  |  | 55.9% | 44. 1% | 100.0% |
|  |  | 35.8% | 31.3% | 33.7% |
|  |  | 18.8% | 14.9% | 33.7% |
| Total | Counting   \| Percentage of drinking  history \| \| --- \| \| Percentage of PHH3  bounded by 13.5 \|   Percentage of total | 53 | 48 | 101 |
|  |  | 52. 5% | 47. 5% | 100.0% |
|  |  | 100.0% | 100.0% | 100.0% |
|  |  | 52. 5% | 47. 5% | 100.0% |

**Chi-square** **test**

|  | value | Degrees  of  freedom | Progressive  significance  (bilateral) | Precise  significance  (bilateral) | Exact  significance  (unilateral) | Point  probabilit  y |
| --- | --- | --- | --- | --- | --- | --- |
| Pearson chi- square Continuity correction Likelihood ratio Fisher precision test  Linear correlation Number of valid cases | 239.  077. 239.  236.  101 | 1  1  1  1 | 625.  781. 625.  627. | 677.  677. 677.  677. | 391.  391. 391.  391. | 149. |

a. 0 cells (0.0%) have an expected count of less than 5. The minimum expected count is 16.16.

b. Calculate for 2x2 tables only

c. Standardized statistics are -.486.

**Symmetrical** **measurement**

|  | value | Progressive significance | Precise significance |
| --- | --- | --- | --- |
| Nominal to Phi | - 049. | 625. | c  . |

| nominal Clem V  Number of column connectio ns  Number of valid cases | 049. | 625. | c  . |
| --- | --- | --- | --- |
|  | 049.  101 | 625. | c  . |

c. Calculations cannot be performed because the temporary file

cannot be opened.

**Alcohol** **history** ***** **HE** **has** **a** **limit** **of** **14.5**

**Crosstabs**

|  | | | HE is bounded by 14.5 | | Total |
| --- | --- | --- | --- | --- | --- |
|  |  |  | 14 or less | 15 or  more |  |
| Drinkin g history | There Count | | 36 | 31 | 67 |
|  | is no Percentage of drinking  history | | 53.7% | 46.3% | 100.0% |
|  | Accounts for the percentage of HE bounded by 14.5 | | 64.3% | 68.9% | 66.3% |
|  | Percentage of total | | 35.6% | 30.7% | 66.3% |
|  | There are | Counting   \| Percentage of drinking history \| \| --- \| \| Percentage of HE bounded by 14.5 \| | 20 | 14 | 34 |
|  |  |  | 58.8% | 41.2% | 100.0% |
|  |  |  | 35.7% | 31. 1% | 33.7% |
|  |  |  | 19.8% | 13.9% | 33.7% |
|  | Percentage of total | |  |  |  |
| Total | Counting   \| Percentage of drinking history \| \| --- \| \| Percentage of HE bounded by 14.5 \|   Percentage of total | | 56 | 45 | 101 |
|  |  |  | 55.4% | 44.6% | 100.0% |
|  |  |  | 100.0% | 100.0% | 100.0% |
|  |  |  | 55.4% | 44.6% | 100.0% |

**Chi-square** **test**

|  | value | Degrees  of  freedom | Progressive  significance  (bilateral) | Precise  significance  (bilateral) | Exact  significance  (unilateral) | Point  probabilit  y |
| --- | --- | --- | --- | --- | --- | --- |
| Pearson chi- square Continuity correction Likelihood ratio Fisher precision test  Linear correlation Number of valid cases | 237.  075. 237.  234.  101 | 1  1  1  1 | 627.  784. 626.  628. | 676.  676. 676.  676. | 393.  393. 393.  393. | 150. |

a. 0 cells (0.0%) have an expected count of less than 5. The minimum expected count is 15.15.

b. Calculate for 2x2 tables only

c. Standardized statistics are -.484.

**Symmetry** **measurement**

|  | | value | Progressive significance | Precise significance |
| --- | --- | --- | --- | --- |
| Nominal to Phi | | - 048. | 627. | c  . |
| nominal | Clem V |  |  |  |
|  |  | 048. | 627. | c  . |
|  |  | 048.  101 | 627. | c  . |
| Number of column connectio ns  Number of valid cases | |  |  |  |

c. Calculations cannot be performed because the temporary file

cannot be opened.

**Alcohol** **history** ***** **KI67** **is** **grouped** **by** **9.5** **percent**

**crosstabs**

|  | | | KI67 is grouped by 9.5 percent | | Total |
| --- | --- | --- | --- | --- | --- |
|  |  |  | 9 or less | 10 or  higher |  |
| Drinkin g history | There Count | | 29 | 38 | 67 |
|  | is no | Percentage of drinking history | 43.3% | 56.7% | 100.0% |
|  | Percentage of KI67 grouped by 9.5 percent | | 67.4% | 65. 5% | 66.3% |
|  | Percentage of total | | 28.7% | 37.6% | 66.3% |
|  | There are | Counting   \| Percentage of drinking history \| \| --- \| \| Percentage of KI67 grouped by 9.5 percent \| | 14 | 20 | 34 |
|  |  |  | 41.2% | 58.8% | 100.0% |
|  |  |  | 32.6% | 34. 5% | 33.7% |
|  |  |  | 13.9% | 19.8% | 33.7% |
|  | Percentage of total | |  |  |  |
| Total | Counting   \| Percentage of drinking history \| \| --- \| \| Percentage of KI67 grouped by 9.5 percent \|   Percentage of total | | 43 | 58 | 101 |
|  |  |  | 42.6% | 57.4% | 100.0% |
|  |  |  | 100.0% | 100.0% | 100.0% |
|  |  |  | 42.6% | 57.4% | 100.0% |

**Chi-square** **test**

|  | value | Degree of freedom | Progressive  significance  (bilateral) | Precise  significance  (bilateral) | Exact  significance  (unilateral) | Point  probabilit  y |
| --- | --- | --- | --- | --- | --- | --- |

| Pearson chi- square Continuity correction Likelihood ratio Fisher precision test  Linear correlation Number of valid cases | 041.  000. 041.  041.  101 | 1  1  1  1 | 840.  1.000 839.  840. | 1.000  1.000 1.000  1.000 | 506.  506. 506.  506. | 165. |
| --- | --- | --- | --- | --- | --- | --- |

a. 0 cells (0.0%) have an expected count of less than 5. The minimum expected count is 14.48.

b. Calculate for 2x2 tables only

c. Standardized statistics are.201.

**Symmetrical** **measurement**

|  | | value | Progressive significance | Precise significance |
| --- | --- | --- | --- | --- |
| Nominal to Phi | | 020. | 840. | c  . |
| nominal | Clem V |  |  |  |
|  |  | 020. | 840. | c  . |
|  |  | 020.  101 | 840. | c  . |
| Number of column connectio ns  Number of valid cases | |  |  |  |

c. Calculations cannot be performed because the temporary file

cannot be opened.

**Alcohol** **history** ***** **P53** **is** **delimited** **by** **50** **percent**

**Crosstabs**

|  | | | P53 is bounded by 50 percent | | Total |
| --- | --- | --- | --- | --- | --- |
|  |  |  | < 50% | 50% or  higher |  |
| Drinkin g history | There is no | Count  Percentage of drinking history  Percentage of P53 bounded by 50 percent | 52 | 15 | 67 |
|  |  |  | 77.6% | 22.4% | 100.0% |
|  |  |  | 65.8% | 68.2% | 66.3% |

| A percentage of the total | | 51. 5% | 14.9% | 66.3% |
| --- | --- | --- | --- | --- |
| There are | Counting   \| Percentage of drinking  history \| \| --- \| \| Percentage of P53 bounded by 50 percent \|   Percentage of total | 27 | 7 | 34 |
|  |  | 79.4% | 20.6% | 100.0% |
|  |  | 34.2% | 31.8% | 33.7% |
|  |  | 26.7% | 6.9% | 33.7% |
| Total | Counting   \| Percentage of drinking  history \| \| --- \| \| Percentage of P53 bounded by 50 percent \|   Percentage of total | 79 | 22 | 101 |
|  |  | 78.2% | 21.8% | 100.0% |
|  |  | 100.0% | 100.0% | 100.0% |
|  |  | 78.2% | 21.8% | 100.0% |

**Chi-square** **test**

|  | value | Degrees  of  freedom | Progressive  significance  (bilateral) | Precise  significance  (bilateral) | Exact  significance  (unilateral) | Point  probabilit  y |
| --- | --- | --- | --- | --- | --- | --- |
| Pearson chi- square Continuity correction Likelihood ratio Fisher precision test  Linear correlation Number of valid cases | 043.  000. 043.  042.  101 | 1  1  1  1 | 836.  1.000 835.  837. | 1.000  1.000 1.000  1.000 | 525.  525. 525.  525. | 198. |

a. 0 cells (0.0%) have an expected count of less than 5. The minimum expected count is 7.41.

b. Calculate for 2x2 tables only

c. Standardized statistics are -.206.

**Symmetrical** **measurement**

|  | value | Progressive significance | Precise significance |
| --- | --- | --- | --- |
| Nominal to Phi | - 021. | 836. | c  . |

| nominal Clem V  Number of column connectio ns  Number of valid cases | 021. | 836. | c  . |
| --- | --- | --- | --- |
|  | 021.  101 | 836. | c  . |

c. Calculations cannot be performed because the temporary file

cannot be opened.

**Smoking** **history** ***** **PHH3** **was** **limited** **to** **13.5**

**crosstabs**

|  | | | PHH3 is bounded by 13.5 | | Total |
| --- | --- | --- | --- | --- | --- |
|  |  |  | 13 or less | Greater than  or equal to  14 |  |
| Smokin g history | There Count | | 35 | 26 | 61 |
|  | is no | Percentage of smoking history | 57.4% | 42.6% | 100.0% |
|  | The percentage of PHH3 bounded by 13.5 | | 66.0% | 54.2% | 60.4% |
|  | Percentage of total | | 34.7% | 25.7% | 60.4% |
|  | There are | Counting   \| Percentage of smoking history \| \| --- \| \| Percentage of PHH3  bounded by 13.5 \| | 18 | 22 | 40 |
|  |  |  | 45.0% | 55.0% | 100.0% |
|  |  |  | 34.0% | 45.8% | 39.6% |
|  |  |  | 17.8% | 21.8% | 39.6% |
|  | Percentage of total | |  |  |  |
| Total | Counting   \| Percentage of smoking history \| \| --- \| \| Percentage of PHH3  bounded by 13.5 \|   Percentage of total | | 53 | 48 | 101 |
|  |  |  | 52. 5% | 47. 5% | 100.0% |
|  |  |  | 100.0% | 100.0% | 100.0% |
|  |  |  | 52. 5% | 47. 5% | 100.0% |

**Chi-square** **test**

|  | value | Degrees  of  freedom | Progressive  significance  (bilateral) | Precise  significance  (bilateral) | Exact  significance  (unilateral) | Point  probabilit  y |
| --- | --- | --- | --- | --- | --- | --- |
| Pearson chi- square Continuity correction Likelihood ratio Fisher precision test  Linear correlation Number of valid cases | 1.484a  1.029 1.486  1.469c  101 | 1  1  1  1 | 223.  310. 223.  225. | 308.  308. 308.  308. | 155.  155. 155.  155. | 078. |

a. 0 cells (0.0%) have an expected count of less than 5. The minimum expected count is 19.01.

b. Calculate for 2x2 tables only

c. 1.212 for standardized statistics.

**Symmetry** **measurement**

|  | | value | Progressive significance | Precise significance |
| --- | --- | --- | --- | --- |
| Nominal to Phi | | 121. | 223. | c  . |
| nominal | Clem V |  |  |  |
|  |  | 121. | 223. | c  . |
|  |  | 120.  101 | 223. | c  . |
| Number of column connectio ns  Number of valid cases | |  |  |  |

c. Calculations cannot be performed because the temporary file

cannot be opened.

**Smoking** **history** ***** **HE** **is** **bounded** **by** **14.5**

**crosstabs**

|  | | | HE is bounded by 14.5 | | Total |
| --- | --- | --- | --- | --- | --- |
|  |  |  | 14 or less | 15 or  more |  |
| Smokin g history | There Count | | 36 | 25 | 61 |
|  | is no Percentage of smoking  history | | 59.0% | 41.0% | 100.0% |
|  | Percentage of HE bounded by 14.5 | | 64.3% | 55.6% | 60.4% |
|  | Percentage of total | | 35.6% | 24.8% | 60.4% |
|  | There are | Counting   \| Percentage of smoking history \| \| --- \| \| Percentage of HE bounded by 14.5 \|   Percentage of total | 20 | 20 | 40 |
|  |  |  | 50.0% | 50.0% | 100.0% |
|  |  |  | 35.7% | 44.4% | 39.6% |
|  |  |  | 19.8% | 19.8% | 39.6% |
| Total | Counting   \| Percentage of smoking history \| \| --- \| \| Percentage of HE bounded by 14.5 \|   Percentage of total | | 56 | 45 | 101 |
|  |  |  | 55.4% | 44.6% | 100.0% |
|  |  |  | 100.0% | 100.0% | 100.0% |
|  |  |  | 55.4% | 44.6% | 100.0% |

**Chi-square** **test**

|  | value | Degree of freedom | Progressive  significance  (bilateral) | Precise  significance  (bilateral) | Exact  significance  (unilateral) | Point  probabilit  y |
| --- | --- | --- | --- | --- | --- | --- |
| Pearson chi-  square | 795. | 1 | 373. | 417. | 246. |  |

| Continuity correction Likelihood ratio Fisher precision test  Linear correlation Number of valid cases | 472. 794.  787.  101 | 1  1  1 | 492. 373.  375. | 417. 417.  417. | 246. 246.  246. | 109. |
| --- | --- | --- | --- | --- | --- | --- |

a. 0 cells (0.0%) have an expected count of less than 5. The minimum expected count is 17.82.

b. Calculate for 2x2 tables only

c. Standardized statistics are.887.

**Symmetrical** **measurement**

|  | | value | Progressive significance | Precise significance |
| --- | --- | --- | --- | --- |
| Nominal to Phi | | 089. | 373. | c  . |
| nominal | Clem V |  |  |  |
|  |  | 089. | 373. | c  . |
|  |  | 088.  101 | 373. | c  . |
| Number of column connectio ns  Number of valid cases | |  |  |  |

c. Calculations cannot be performed because the temporary file

cannot be opened.

**Smoking** **history** ***** **KI67** **grouped** **by** **9.5** **percent**

**crosstabs**

|  | | | KI67 is grouped by 9.5 percent | | Total |
| --- | --- | --- | --- | --- | --- |
|  |  |  | 9 or less | 10 or  higher |  |
| Smokin g history | There is no | count  Percentage of smoking history  Percentage of KI67 grouped by 9.5 percent | 31 | 30 | 61 |
|  |  |  | 50.8% | 49.2% | 100.0% |
|  |  |  | 72. 1% | 51.7% | 60.4% |

| Percentage of total | | 30.7% | 29.7% | 60.4% |
| --- | --- | --- | --- | --- |
| There are | Counting   \| Percentage of smoking history \| \| --- \| \| Percentage of KI67 grouped by 9.5 percent \|   Percentage of total | 12 | 28 | 40 |
|  |  | 30.0% | 70.0% | 100.0% |
|  |  | 27.9% | 48.3% | 39.6% |
|  |  | 11.9% | 27.7% | 39.6% |
| Total | Counting   \| Percentage of smoking history \| \| --- \| \| Percentage of KI67 grouped by 9.5 percent \|   Percentage of total | 43 | 58 | 101 |
|  |  | 42.6% | 57.4% | 100.0% |
|  |  | 100.0% | 100.0% | 100.0% |
|  |  | 42.6% | 57.4% | 100.0% |

**Chi-square** **test**

|  | value | Degrees  of  freedom | Progressive  significance  (bilateral) | Precise  significance  (bilateral) | Exact  significance  (unilateral) | Point  probabilit  y |
| --- | --- | --- | --- | --- | --- | --- |
| Pearson chi- square Continuity correction Likelihood ratio Fisher precision test  Linear correlation Number of valid cases | 4.283a  3.474 4.363  4.241c  101 | 1  1  1  1 | 038.  062. 037.  039. | 043.  043. 043.  043. | 030.  030. 030.  030. | 020. |

a. 0 cells (0.0%) have an expected count of less than 5. The minimum expected count is 17.03.

b. Calculate for 2x2 tables only

c. Standardized statistics are 2.059.

**Symmetrical** **measurement**

|  | value | Progressive significance | Precise significance |
| --- | --- | --- | --- |
| Nominal to Phi | 206. | 038. | c  . |

| nominal Clem V  Number of column connectio ns  Number of valid cases | 206. | 038. | c  . |
| --- | --- | --- | --- |
|  | 202.  101 | 038. | c  . |

c. Calculations cannot be performed because the temporary file

cannot be opened.

**Smoking** **history** ***** **P53** **is** **limited** **by** **50** **percent**

**Crosstabs**

|  | | | P53 is bounded by 50 percent | | Total |
| --- | --- | --- | --- | --- | --- |
|  |  |  | < 50% | 50% or  higher |  |
| Smokin g history | There Count | | 47 | 14 | 61 |
|  | is no | Percentage of smoking history | 77.0% | 23.0% | 100.0% |
|  | Percentage of P53 bounded by 50 percent | | 59. 5% | 63.6% | 60.4% |
|  | A percentage of the total | | 46. 5% | 13.9% | 60.4% |
|  | There are | Counting   \| Percentage of smoking history \| \| --- \| \| Percentage of P53 bounded by 50 percent \| | 32 | 8 | 40 |
|  |  |  | 80.0% | 20.0% | 100.0% |
|  |  |  | 40. 5% | 36.4% | 39.6% |
|  |  |  | 31.7% | 7.9% | 39.6% |
|  | Percentage of total | |  |  |  |
| Total | Counting   \| Percentage of smoking history \| \| --- \| \| Percentage of P53 bounded by 50 percent \|   Percentage of total | | 79 | 22 | 101 |
|  |  |  | 78.2% | 21.8% | 100.0% |
|  |  |  | 100.0% | 100.0% | 100.0% |
|  |  |  | 78.2% | 21.8% | 100.0% |

**Chi-square** **test**

|  | value | Degrees  of  freedom | Progressive  significance  (bilateral) | Precise  significance  (bilateral) | Exact  significance  (unilateral) | Point  probabilit  y |
| --- | --- | --- | --- | --- | --- | --- |
| Pearson chi- square Continuity correction Likelihood ratio Fisher precision test  Linear correlation Number of valid cases | 123.  011. 124.  122.  101 | 1  1  1  1 | 725.  916. 724.  727. | 808.  808. 808.  808. | 462.  462. 462.  462. | 185. |

a. 0 cells (0.0%) have an expected count of less than 5. The minimum expected count is 8.71.

b. Calculate for 2x2 tables only

c. Standardized statistics are -.350.

**Symmetrical** **measurement**

|  | | value | Progressive significance | Precise significance |
| --- | --- | --- | --- | --- |
| Nominal to Phi | | - 035. | 725. | c  . |
| nominal | Clem V |  |  |  |
|  |  | 035. | 725. | c  . |
|  |  | 035.  101 | 725. | c  . |
| Number of column connectio ns  Number of valid cases | |  |  |  |

c. Calculations cannot be performed because the temporary file

cannot be opened.

**Tumor** **volume** ***** **PHH3** **was** **limited** **by** **13.5**

**crosstabs**

|  | | PHH3 is bounded by 13.5 | | Total |
| --- | --- | --- | --- | --- |
|  |  | 13 or less | Greater than  or equal to  14 |  |
| Mass Less than 4 Counting | | 47 | 33 | 80 |
|  | |  |  |  |
| volume | Percentage of the volume of the mass | 58.8% | 41.3% | 100.0% |
| The percentage of PHH3 bounded by 13.5 | | 88.7% | 68.8% | 79.2% |
| Percentage of total   \| Less than 15 \| Counting \| \| --- \| --- \| \| Percentage of the volume of the mass \| \| Percentage of PHH3  bounded by 13.5 \| \| Percentage of total \|   15 or greater Count   \| Percentage of the volume of the mass \| \| --- \| \| Percentage of PHH3  bounded by 13.5 \| | | 46. 5% | 32.7% | 79.2% |
|  |  | 5 | 8 | 13 |
|  |  | 38. 5% | 61. 5% | 100.0% |
|  |  | 9.4% | 16.7% | 12.9% |
|  |  | 5.0% | 7.9% | 12.9% |
|  |  | 1 | 7 | 8 |
|  |  | 12. 5% | 87. 5% | 100.0% |
|  |  | 1.9% | 14.6% | 7.9% |
|  |  | 1.0% | 6.9% | 7.9% |
| Percentage of total | |  |  |  |
| Total | Counting   \| Percentage of the volume of the mass \| \| --- \| \| Percentage of PHH3  bounded by 13.5 \|   Percentage of total | 53 | 48 | 101 |
|  |  | 52. 5% | 47. 5% | 100.0% |
|  |  | 100.0% | 100.0% | 100.0% |
|  |  | 52. 5% | 47. 5% | 100.0% |

**Chi-square** **test**

|  | value | Degrees  of  freedom | Progressive  significance  (bilateral) | Precise  significance  (bilateral) | Accuracy  significance  (unilateral) | Point  probabilit  y |
| --- | --- | --- | --- | --- | --- | --- |
| Pearson chi- square Likelihood ratio Fisher precision test  Linear correlation Number of valid cases | 7.413a  7.976  b  .  7.311c  101 | 2  2  1 | 025. 019.  007. | b  .  b  .  b  .  007. | 005. | 003. |

a. 2 cells (33.3%) have an expected count of less than 5. The minimum expected count is 3.80.

b. The calculation cannot be performed because the temporary file cannot be opened.

c. Standardized statistics are 2.704.

**Symmetrical** **measurement**

|  | | value | Progressive significance | Precise significance |
| --- | --- | --- | --- | --- |
| Nominal to Phi | | 271. | 025. | c  . |
| nominal | Clem V |  |  |  |
|  |  | 271. | 025. | c  . |
|  |  | 261.  101 | 025. | c  . |
| Number of column connectio ns  Number of valid cases | |  |  |  |

c. Calculations cannot be performed because the temporary file

cannot be opened.

**Volume** **of** **mass** ***** **HE** **is** **bounded** **by** **14.5**

**crosstab**

|  | | HE is bounded by 14.5 | | Total |
| --- | --- | --- | --- | --- |
|  |  | 14 or less | 15 or  more |  |
| Mass Less than 4 Counting | | 49 | 31 | 80 |
| volume | \| Percentage of the volume of the mass \| \| --- \| \| Percentage of HE bounded by 14.5 \| |  |  |  |
|  |  | 61.3% | 38.8% | 100.0% |
|  |  | 87. 5% | 68.9% | 79.2% |
|  |  | 48. 5% | 30.7% | 79.2% |
| Percentage of total | |  |  |  |

| Less than 15 Counting  Percentage of the volume of the mass  Accounts for the percentage of HE bounded by 14.5  Percentage of total  15 or greater Count   \| Percentage of the volume of the mass \| \| --- \| \| Percentage of HE bounded by 14.5 \|   Percentage of total | | 6 | 7 | 13 |
| --- | --- | --- | --- | --- | --- | --- |
|  |  | 46.2% | 53.8% | 100.0% |
|  |  | 10.7% | 15.6% | 12.9% |
|  |  | 5.9% | 6.9% | 12.9% |
|  |  | 1 | 7 | 8 |
|  |  | 12. 5% | 87. 5% | 100.0% |
|  |  | 1.8% | 15.6% | 7.9% |
|  |  | 1.0% | 6.9% | 7.9% |
| Total | Counting   \| Percentage of the volume of the mass \| \| --- \| \| Percentage of HE bounded by 14.5 \|   Percentage of total | 56 | 45 | 101 |
|  |  | 55.4% | 44.6% | 100.0% |
|  |  | 100.0% | 100.0% | 100.0% |
|  |  | 55.4% | 44.6% | 100.0% |

**Chi-square** **test**

|  | value | Degrees  of  freedom | Progressive  significance  (bilateral) | Precise  significance  (bilateral) | Exact  significance  (unilateral) | Point  probabilit  y |
| --- | --- | --- | --- | --- | --- | --- |
| Pearson chi- square Likelihood ratio Fisher precision test  Linear correlation Number of valid cases | 7.518a  8.024  b  .  7.134c  101 | 2  2  1 | 023. 018.  008. | b  .  b  .  b  .  008. | 006. | 004. |

a. 2 cells (33.3%) have an expected count of less than 5. The minimum expected count is 3.56.

b. The calculation cannot be performed because the temporary file cannot be opened.

c. Standardized statistics are 2.671.

**Symmetrical** **measurement**

|  | | value | Progressive significance | Precise significance |
| --- | --- | --- | --- | --- |
| Nominal to Phi | | 273. | 023. | c  . |
| nominal | Clem V |  |  |  |
|  |  | 273. | 023. | c  . |
|  |  | 263.  101 | 023. | c  . |
| Number of column connectio ns  Number of valid cases | |  |  |  |

c. Calculations cannot be performed because the temporary file

cannot be opened.

**Volume** **of** **mass** ***** **KI67** **grouped** **by** **9.5** **percent**

**crosstabs**

|  | | KI67 is grouped by 9.5 percent | | Total |
| --- | --- | --- | --- | --- |
|  |  | 9 or less | 10 or  higher |  |
| Mass Less than 4 Counting | | 40 | 40 | 80 |
|  | |  |  |  |
| volume | Percentage of the volume of the mass | 50.0% | 50.0% | 100.0% |
| Percentage of KI67 grouped by 9.5 percent | | 93.0% | 69.0% | 79.2% |
| Percentage of total   \| Less than 15 \| Counting \| \| --- \| --- \| \| Percentage of the volume of the mass \| \| Percentage of KI67 grouped by 9.5 percent \| \| Percentage of total \|   15 or greater Count   \| Percentage of the volume of the mass \| \| --- \| \| Percentage of KI67 grouped by 9.5 percent \| | | 39.6% | 39.6% | 79.2% |
|  |  | 2 | 11 | 13 |
|  |  | 15.4% | 84.6% | 100.0% |
|  |  | 4.7% | 19.0% | 12.9% |
|  |  | 2.0% | 10.9% | 12.9% |
|  |  | 1 | 7 | 8 |
|  |  | 12. 5% | 87. 5% | 100.0% |
|  |  | 2.3% | 12. 1% | 7.9% |
|  |  | 1.0% | 6.9% | 7.9% |
| Percentage of total | |  |  |  |
| Total | count   \| Percentage of the volume of the mass \| \| --- \| \| Percentage of KI67 grouped by 9.5 percent \|   Percentage of total | 43 | 58 | 101 |
|  |  | 42.6% | 57.4% | 100.0% |
|  |  | 100.0% | 100.0% | 100.0% |
|  |  | 42.6% | 57.4% | 100.0% |

**Chi-square** **test**

|  | value | Degrees  of  freedom | Progressive  significance  (bilateral) | Precise  significance  (bilateral) | Exact  significance  (unilateral) | Point  probabilit  y |
| --- | --- | --- | --- | --- | --- | --- |

| Pearson chi- square Likelihood ratio Fisher precision test  Linear correlation Number of valid cases | 8.695a  9.685  b  .  7.693c  101 | 2  2  1 | 013. 008.  006. | b  .  b  .  b  .  006. | 003. | 002. |
| --- | --- | --- | --- | --- | --- | --- |

a. 2 cells (33.3%) have an expected count of less than 5. The minimum expected count is 3.41.

b. The calculation cannot be performed because the temporary file cannot be opened.

c. Standardized statistics are 2.774.

**Symmetry** **measurement**

|  | | value | Progressive significance | Precise significance |
| --- | --- | --- | --- | --- |
| Nominal to Phi | | 293. | 013. | c  . |
| nominal | Clem V |  |  |  |
|  |  | 293. | 013. | c  . |
|  |  | 282.  101 | 013. | c  . |
| Number of column connectio ns  Number of valid cases | |  |  |  |

c. Calculations cannot be performed because the temporary file

cannot be opened.

**Volume** **of** **the** **tumor** ***** **P53** **is** **limited** **by** **50** **percent**

**crosstabs**

|  | | | P53 is bounded by 50 percent | | Total |
| --- | --- | --- | --- | --- | --- |
|  |  |  | < 50% | 50% or  higher |  |
| Mass  volume | Less than 4 | Counting | 67 | 13 | 80 |
|  |  | Percentage of the volume of the mass | 83.8% | 16.3% | 100.0% |
|  |  | Percentage of P53 bounded by 50 percent | 84.8% | 59. 1% | 79.2% |

| Percentage of total   \| Less than 15 \| Counting \| \| --- \| --- \| \| Percentage of the volume of the mass \| \| Percentage of P53 bounded by 50 percent \| \| Percentage of total \|   15 or greater Count   \| Percentage of the volume of the mass \| \| --- \| \| Percentage of P53 bounded by 50 percent \|   Percentage of total | | 66.3% | 12.9% | 79.2% |
| --- | --- | --- | --- | --- | --- | --- | --- | --- | --- | --- | --- |
|  |  | 7 | 6 | 13 |
|  |  | 53.8% | 46.2% | 100.0% |
|  |  | 8.9% | 27.3% | 12.9% |
|  |  | 6.9% | 5.9% | 12.9% |
|  |  | 5 | 3 | 8 |
|  |  | 62. 5% | 37. 5% | 100.0% |
|  |  | 6.3% | 13.6% | 7.9% |
|  |  | 5.0% | 3.0% | 7.9% |
| Total | Counting   \| Percentage of the volume of the mass \| \| --- \| \| Percentage of P53 bounded by 50 percent \|   Percentage of total | 79 | 22 | 101 |
|  |  | 78.2% | 21.8% | 100.0% |
|  |  | 100.0% | 100.0% | 100.0% |
|  |  | 78.2% | 21.8% | 100.0% |

**Chi-square** **test**

|  | value | Degrees  of  freedom | Progressive  significance  (bilateral) | Precise  significance  (bilateral) | Exact  significance  (unilateral) | Point  probabilit  y |
| --- | --- | --- | --- | --- | --- | --- |
| Pearson chi- square Likelihood ratio Fisher's exact test  Linear correlation Number of valid cases | 7.129a  6.339  b  .  5.118c  101 | 2  2  1 | 028. 042.  024. | b  .  b  .  b  .  028. | 025. | 015. |

a. 2 cells (33.3%) have an expected count of less than 5. The minimum expected count is 1.74.

b. The calculation cannot be performed because the temporary file cannot be opened.

c. 2.262 for standardized statistics.

**Symmetrical** **measurement**

|  | | value | Progressive significance | Precise significance |
| --- | --- | --- | --- | --- |
| Nominal to Phi | | 266. | 028. | c  . |
| nominal | Clem V |  |  |  |
|  |  | 266. | 028. | c  . |
|  |  | 257.  101 | 028. | c  . |
| Number of column connectio ns  Number of valid cases | |  |  |  |

c. Calculations cannot be performed because the temporary file

cannot be opened.

**Relapse** **or** **not** ***** **PHH3** **is** **bounded** **by** **13.5**

**crosstabs**

|  | | | PHH3 is bounded by 13.5 | | Total |
| --- | --- | --- | --- | --- | --- |
|  |  |  | 13 or less | Greater than  or equal to  14 |  |
| Relapse or not | There is no | Count  Percentage of whether or not it has recurred  Percentage of PHH3  bounded by 13.5 | 47 | 23 | 70 |
|  |  |  | 67. 1% | 32.9% | 100.0% |
|  |  |  | 88.7% | 47.9% | 69.3% |
| Percentage of total | | |  |  |  |
|  |  |  | 46. 5% | 22.8% | 69.3% |
|  |  |  | 6 | 25 | 31 |
| There are | | Counting   \| Percentage of recurrence or not \| \| --- \| \| Percentage of PHH3  bounded by 13.5 \|   Percentage of total |  |  |  |
|  |  |  | 19.4% | 80.6% | 100.0% |
|  |  |  | 11.3% | 52. 1% | 30.7% |
|  |  |  | 5.9% | 24.8% | 30.7% |
| Total | Counting   \| Percentage of whether or not there is a relapse \| \| --- \| \| Percentage of PHH3  bounded by 13.5 \|   Percentage of total | | 53 | 48 | 101 |
|  |  |  | 52. 5% | 47. 5% | 100.0% |
|  |  |  | 100.0% | 100.0% | 100.0% |
|  |  |  | 52. 5% | 47. 5% | 100.0% |

**Chi-square** **test**

|  | value | Degrees  of  freedom | Progressive  significance  (bilateral) | Precise  significance  (bilateral) | Exact  significance  (unilateral) | Point  probabilit  y |
| --- | --- | --- | --- | --- | --- | --- |
| Pearson chi- square Continuity correction Likelihood ratio Fisher precision test | 19.674a  17.805 20.663 | 1  1  1 | 000.  000. 000. | 000.  000.  000. | 000.  000.  000. |  |

a. The expected count of 0 cells (0.0%) is less than 5. The minimum expected count is 14.73.

b. Calculate for 2x2 tables only

c. 4.414 for standardized statistics.

**Symmetrical** **measurement**

|  | | value | Progressive significance | Precise significance |
| --- | --- | --- | --- | --- |
| Nominal to Phi | | 441. | 000. | c  . |
| nominal | Clem V |  |  |  |
|  |  | 441. | 000. | c  . |
|  |  | 404.  101 | 000. | c  . |
| Column  linkage  number Number of valid cases | |  |  |  |

c. Calculations cannot be performed because the temporary file

cannot be opened.

**Relapse** **or** **not** ***** **HE** **is** **bounded** **by** **14.5**

**Crosstabs**

|  | | | HE is bounded by 14.5 | | Total |
| --- | --- | --- | --- | --- | --- |
|  |  |  | 14 or less | 15 or  more |  |
| Relapse or not | There is no | Count   \| Percentage of whether or not it has recurred \| \| --- \| \| Percentage of HE bounded by 14.5 \|   Percentage of total | 49 | 21 | 70 |
|  |  |  | 70.0% | 30.0% | 100.0% |
|  |  |  | 87. 5% | 46.7% | 69.3% |
|  |  |  | 48. 5% | 20.8% | 69.3% |

| There are | count   \| Percentage of whether or not it has relapsed \| \| --- \| \| Percentage of HE bounded by 14.5 \|   Percentage of total | 7 | 24 | 31 |
| --- | --- | --- | --- | --- | --- | --- |
|  |  | 22.6% | 77.4% | 100.0% |
|  |  | 12. 5% | 53.3% | 30.7% |
|  |  | 6.9% | 23.8% | 30.7% |
| total | Counting   \| Percentage of whether or not there is a relapse \| \| --- \| \| Percentage of HE bounded by 14.5 \|   Percentage of total | 56 | 45 | 101 |
|  |  | 55.4% | 44.6% | 100.0% |
|  |  | 100.0% | 100.0% | 100.0% |
|  |  | 55.4% | 44.6% | 100.0% |

**Chi-square** **test**

|  | value | Degrees  of  freedom | Progressive  significance  (bilateral) | Precise  significance  (bilateral) | Exact  significance  (unilateral) | Point  probabilit  y |
| --- | --- | --- | --- | --- | --- | --- |
| Pearson chi- square Continuity correction Likelihood ratio Fisher precision test  Linear correlation Number of valid cases | 19.557a  17.684 20.176  19.363c  101 | 1  1  1  1 | 000.  000. 000.  000. | 000.  000. 000.  000. | 000.  000. 000.  000. | 000. |

a. 0 cells (0.0%) have an expected count of less than 5. The minimum expected count is 13.81.

b. Calculate for 2x2 tables only

c. 4.400 for standardized statistics.

**Symmetrical** **measurement**

|  | | value | Progressive significance | Precise significance |
| --- | --- | --- | --- | --- |
| Nominal to nominal | Phi  Klem V | 440. | 000. | c  . |
|  |  | 440. | 000. | c  . |

| Number of column connectio ns  Number of valid cases | 403.  101 | 000. | c  . |
| --- | --- | --- | --- |

c. Calculations cannot be performed because the temporary file

cannot be opened.

**Relapse** **or** **not** ***** **KI67** **grouped** **by** **9.5** **percent**

|  | | KI67 is grouped by 9.5 percent | | Total |
| --- | --- | --- | --- | --- |
|  |  | 9 or less | 10 or  higher |  |
| Relapse or There Count  not is no Percentage of whether or  not it has recurred  Percentage of KI67 grouped by 9.5 percent  Percentage of total | | 40 | 30 | 70 |
|  |  | 57. 1% | 42.9% | 100.0% |
|  |  | 93.0% | 51.7% | 69.3% |
|  |  | 39.6% | 29.7% | 69.3% |
|  |  | 3 | 28 | 31 |
| There are | Counting   \| Percentage of whether or not it has relapsed \| \| --- \| \| Percentage of KI67 grouped by 9.5 percent \|   A percentage of the total |  |  |  |
|  |  | 9.7% | 90.3% | 100.0% |
|  |  | 7.0% | 48.3% | 30.7% |
|  |  | 3.0% | 27.7% | 30.7% |
| Total | Counting   \| Percentage of whether or not there is a relapse \| \| --- \| \| Percentage of KI67 grouped by 9.5 percent \|   Percentage of total | 43 | 58 | 101 |
|  |  | 42.6% | 57.4% | 100.0% |
|  |  | 100.0% | 100.0% | 100.0% |
|  |  | 42.6% | 57.4% | 100.0% |

**Chi-square** **test**

|  | value | Degrees  of  freedom | Progressive  significance  (bilateral) | Precise  significance  (bilateral) | Exact  significance  (unilateral) | Point  probabilit  y |
| --- | --- | --- | --- | --- | --- | --- |
| Pearson chi- square Continuity correction Likelihood ratio Fisher precision test  Linear correlation Number of valid cases | 19.799a  17.905 22.461  19.603c  101 | 1  1  1  1 | 000.  000. 000.  000. | 000.  000. 000.  000. | 000.  000. 000.  000. | 000. |

a. 0 cells (0.0%) have an expected count of less than 5. The minimum expected count is 13.20.

b. Calculate for 2x2 tables only

c. 4.428 for standardized statistics.

**Symmetrical** **measurement**

|  | | value | Progressive significance | Precise significance |
| --- | --- | --- | --- | --- |
| Nominal to Phi | | 443. | 000. | c  . |
| nominal | Clem V |  |  |  |
|  |  | 443. | 000. | c  . |
|  |  | 405.  101 | 000. | c  . |
| Number of column connectio ns  Number of valid cases | |  |  |  |

c. Calculations cannot be performed because the temporary file

cannot be opened.

**Relapse** **or** **not** ***** **P53** **is** **bounded** **by** **50** **percent**

**crosstab**

|  | | P53 is bounded by 50 percent | | Total |
| --- | --- | --- | --- | --- |
|  |  | < 50% | 50% or  higher |  |
| Relapse or There Count  not is no Percentage of whether or  not it has recurred  Percentage of P53 bounded by 50 percent  Percentage of total | | 57 | 13 | 70 |
|  |  | 81.4% | 18.6% | 100.0% |
|  |  | 72.2% | 59. 1% | 69.3% |
|  |  | 56.4% | 12.9% | 69.3% |
|  |  | 22 | 9 | 31 |
| There are | Counting   \| Percentage of whether or not it has relapsed \| \| --- \| \| Accounts for the percentage of P53 bounded by 50 percent \|   Percentage of total |  |  |  |
|  |  | 71.0% | 29.0% | 100.0% |
|  |  | 27.8% | 40.9% | 30.7% |
|  |  | 21.8% | 8.9% | 30.7% |
| Total | Counting   \| Percentage of whether or not there is a relapse \| \| --- \| \| Percentage of P53 bounded by 50 percent \|   Percentage of total | 79 | 22 | 101 |
|  |  | 78.2% | 21.8% | 100.0% |
|  |  | 100.0% | 100.0% | 100.0% |
|  |  | 78.2% | 21.8% | 100.0% |

**Chi-square** **test**

|  | value | Degrees  of  freedom | Progressive  significance  (bilateral) | Precise  significance  (bilateral) | Exact  significance  (unilateral) | Point  probabilit  y |
| --- | --- | --- | --- | --- | --- | --- |

| Pearson chi- square Continuity correction Likelihood ratio Fisher's exact test  Linear correlation Number of valid cases | 1.380a  834. 1.332  1.366c  101 | 1  1  1  1 | 240.  361. 249.  242. | 297.  297. 297.  297. | 180.  180. 180.  180. | 102. |
| --- | --- | --- | --- | --- | --- | --- |

a. 0 cells (0.0%) have an expected count of less than 5. The minimum expected count is 6.75.

b. Calculate for 2x2 tables only

c. 1.169 for standardized statistics.

**Symmetrical** **measurement**

|  | | value | Progressive significance | Precise significance |
| --- | --- | --- | --- | --- |
| Nominal to Phi | | 117. | 240. | c  . |
| nominal | Clem V |  |  |  |
|  |  | 117. | 240. | c  . |
|  |  | 116.  101 | 240. | c  . |
| Number of column connectio ns  Number of valid cases | |  |  |  |

c. Calculations cannot be performed because the temporary file

cannot be opened.

**Dead** **or** **not** ***** **PHH3** **is** **bounded** **by** **13.5**

**crosstabs**

|  | | | PHH3 is bounded by 13.5 | | Total |
| --- | --- | --- | --- | --- | --- |
|  |  |  | 13 or less | Greater than  or equal to  14 |  |
| Dead or not | no | Count  Percentage of whether dead or not  Percentage of PHH3  bounded by 13.5 | 52 | 41 | 93 |
|  |  |  | 55.9% | 44. 1% | 100.0% |
|  |  |  | 98. 1% | 85.4% | 92. 1% |

| Percentage of total | | 51. 5% | 40.6% | 92. 1% |
| --- | --- | --- | --- | --- |
|  | |  |  |  |
| is | Count   \| Percentage of death or not \| \| --- \| \| Percentage of PHH3  bounded by 13.5 \| | 1 | 7 | 8 |
|  |  | 12. 5% | 87. 5% | 100.0% |
|  |  | 1.9% | 14.6% | 7.9% |
|  |  | 1.0% | 6.9% | 7.9% |
| Percentage of total | |  |  |  |
| Total | Counting   \| Percentage of whether dead or not \| \| --- \| \| Percentage of PHH3  bounded by 13.5 \|   Percentage of total | 53 | 48 | 101 |
|  |  | 52. 5% | 47. 5% | 100.0% |
|  |  | 100.0% | 100.0% | 100.0% |
|  |  | 52. 5% | 47. 5% | 100.0% |

**Chi-square** **test**

|  | value | Degrees  of  freedom | Progressive  significance  (bilateral) | Precise  significance  (bilateral) | Exact  significance  (unilateral) | Point  probabilit  y |
| --- | --- | --- | --- | --- | --- | --- |
| Pearson chi- square Continuity correction Likelihood ratio Fisher precision test  Linear correlation Number of valid cases | 5.567a  3.962 6.119  5.512c  101 | 1  1  1  1 | 018.  047. 013.  019. | 026.  026. 026.  026. | 021.  021. 021.  021. | 019. |

a. 2 cells (50.0%) have an expected count less than 5. The minimum expected count is 3.80.

b. Calculate for 2x2 tables only

c. 2.348 for standardized statistics.

**Symmetrical** **measurement**

|  | | value | Progressive significance | Precise significance |
| --- | --- | --- | --- | --- |
| Nominal to nominal | Phi  Clem V | 235. | 018. | c  . |
|  |  | 235. | 018. | c  . |

| Column  linkage  number Number of valid cases | 229.  101 | 018. | c  . |
| --- | --- | --- | --- |

c. Calculations cannot be performed because the temporary file

cannot be opened.

**Dead** **or** **not** ***** **HE** **is** **bounded** **by** **14.5**

**crosstabs**

|  | HE is bounded by 14.5 | Total |
| --- | --- | --- |

|  | | | 14 or less | 15 or  more |  |
| --- | --- | --- | --- | --- | --- |
| Dead or not | no Count | | 55 | 38 | 93 |
|  | Percentage of whether dead or not | | 59. 1% | 40.9% | 100.0% |
|  | Percentage of HE bounded by 14.5 | | 98.2% | 84.4% | 92. 1% |
|  | Percentage of total | | 54. 5% | 37.6% | 92. 1% |
|  | is | count   \| Percentage of whether  dead or not \| \| --- \| \| Percentage of HE bounded by 14.5 \| | 1 | 7 | 8 |
|  |  |  | 12. 5% | 87. 5% | 100.0% |
|  |  |  | 1.8% | 15.6% | 7.9% |
|  |  |  | 1.0% | 6.9% | 7.9% |
|  | Percentage of total | |  |  |  |
| total | Counting   \| Percentage of whether  dead or not \| \| --- \| \| Percentage of HE bounded by 14.5 \|   Percentage of total | | 56 | 45 | 101 |
|  |  |  | 55.4% | 44.6% | 100.0% |
|  |  |  | 100.0% | 100.0% | 100.0% |
|  |  |  | 55.4% | 44.6% | 100.0% |

**Chi-square** **test**

|  | value | Degrees  of  freedom | Progressive  significance  (bilateral) | Precise  significance  (bilateral) | Exact  significance  (unilateral) | Point  probabilit  y |
| --- | --- | --- | --- | --- | --- | --- |
| Pearson chi- square Continuity correction Likelihood ratio Fisher precision test  Linear correlation Number of valid cases | 6.486a  4.736 6.987  6.422c  101 | 1  1  1  1 | 011.  030. 008.  011. | 021.  021. 021.  021. | 014.  014. 014.  014. | 013. |

a. 2 cells (50.0%) have an expected count of less than 5. The minimum expected count is 3.56.

b. Calculate for 2x2 tables only

c. 2.534 in standardized statistics.

**Symmetrical** **measurement**

|  | | value | Progressive significance | Precise significance |
| --- | --- | --- | --- | --- |
| Nominal to Phi | | 253. | 011. | c  . |
| nominal | Clem V |  |  |  |
|  |  | 253. | 011. | c  . |
|  |  | 246.  101 | 011. | c  . |
| Number of column connectio ns  Number of valid cases | |  |  |  |

c. Calculations cannot be performed because the temporary file

cannot be opened.

**Dead** **or** **not** ***** **KI67** **grouped** **by** **9.5** **percent**

**crosstabs**

|  | | | KI67 is grouped by 9.5 percent | | Total |
| --- | --- | --- | --- | --- | --- |
|  |  |  | 9 or less | 10 or  higher |  |
| Dead or not | no Count | | 43 | 50 | 93 |
|  | Percentage of whether dead or not | | 46.2% | 53.8% | 100.0% |
|  | Percentage of KI67 grouped by 9.5 percent | | 100.0% | 86.2% | 92. 1% |
|  | Percentage of total | | 42.6% | 49. 5% | 92. 1% |
|  | is | Count   \| Percentage of whether  dead or not \| \| --- \| \| Percentage of KI67 grouped by 9.5 percent \| | 0 | 8 | 8 |
|  |  |  | 0.0% | 100.0% | 100.0% |
|  |  |  | 0.0% | 13.8% | 7.9% |
|  |  |  | 0.0% | 7.9% | 7.9% |
|  | Percentage of total | |  |  |  |
| Total | Counting   \| Percentage of whether  dead or not \| \| --- \| \| Percentage of KI67 grouped by 9.5 percent \|   Percentage of total | | 43 | 58 | 101 |
|  |  |  | 42.6% | 57.4% | 100.0% |
|  |  |  | 100.0% | 100.0% | 100.0% |
|  |  |  | 42.6% | 57.4% | 100.0% |

**Chi-square** **test**

|  | value | Degrees  of  freedom | Progressive  significance  (bilateral) | Precise  significance  (bilateral) | Exact  significance  (unilateral) | Point  probabilit  y |
| --- | --- | --- | --- | --- | --- | --- |
| Pearson chi- square Continuity correction Likelihood ratio | 6.441a  4.689  9.382 | 1  1  1 | 011.  030.  002. | 019.  010. | 009.  009. |  |

| Fisher precision test  Linear correlation Number of valid cases | 6.377c  101 | 1 | 012. | 019.  019. | 009.  009. | 009. |
| --- | --- | --- | --- | --- | --- | --- |

a. 2 cells (50.0%) have an expected count of less than 5. The minimum expected count is 3.41.

b. Calculate for 2x2 tables only

c. 2.525 for standardized statistics.

**Symmetrical** **measurement**

|  | | value | Progressive significance | Precise significance |
| --- | --- | --- | --- | --- |
| Nominal to Phi | | 253. | 011. | c  . |
| nominal | Clem V |  |  |  |
|  |  | 253. | 011. | c  . |
|  |  | 245.  101 | 011. | c  . |
| Number of column connectio ns  Number of valid cases | |  |  |  |

c. Calculations cannot be performed because the temporary file

cannot be opened.

**Dead** **or** **not** ***** **P53** **is** **bounded** **by** **50** **percent**

**Crosstabs**

|  | | | P53 is bounded by 50 percent | | Total |
| --- | --- | --- | --- | --- | --- |
|  |  |  | < 50% | 50% or  higher |  |
| Dead or not | no | Count  Percentage of whether dead or not  Percentage of P53 bounded by 50 percent | 74 | 19 | 93 |
|  |  |  | 79.6% | 20.4% | 100.0% |
|  |  |  | 93.7% | 86.4% | 92. 1% |

| Percentage of total | | 73.3% | 18.8% | 92. 1% |
| --- | --- | --- | --- | --- |
|  | |  |  |  |
| is | Count   \| Percentage of whether dead or not \| \| --- \| \| Percentage of P53 bounded by 50 percent \| | 5 | 3 | 8 |
|  |  | 62. 5% | 37. 5% | 100.0% |
|  |  | 6.3% | 13.6% | 7.9% |
|  |  | 5.0% | 3.0% | 7.9% |
| Percentage of total | |  |  |  |
| Total | Counting   \| Percentage of whether dead or not \| \| --- \| \| Percentage of P53 bounded by 50 percent \|   Percentage of total | 79 | 22 | 101 |
|  |  | 78.2% | 21.8% | 100.0% |
|  |  | 100.0% | 100.0% | 100.0% |
|  |  | 78.2% | 21.8% | 100.0% |

**Chi-square** **test**

|  | value | Degrees  of  freedom | Progressive  significance  (bilateral) | Precise  significance  (bilateral) | Exact  significance  (unilateral) | Point  probabilit  y |
| --- | --- | --- | --- | --- | --- | --- |
| Pearson chi- square Continuity correction Likelihood ratio Fisher precision test  Linear correlation Number of valid cases | 1.260a  457. 1.118  1.247c  101 | 1  1  1  1 | 262.  499. 290.  264. | 367.  367. 367.  367. | 238.  238. 238.  238. | 172. |

a. 1 cell (25.0%) has an expected count of less than 5. The minimum expected count is 1.74.

b. Calculate for 2x2 tables only

c. 1.117 for standardized statistics.

**Symmetrical** **measurement**

|  | value | Progressive significance | Precise significance |
| --- | --- | --- | --- |
| Nominal to Phi | 112. | 262. | c  . |

| nominal Clem V  Number of column connectio ns  Number of valid cases | 112. | 262. | c  . |
| --- | --- | --- | --- |
|  | 111.  101 | 262. | c  . |

c. Calculations cannot be performed because the temporary file

cannot be opened.

**Infiltrating** **depth** ***** **PHH3** **is** **bounded** **by** **13.5**

**crosstabs**

|  | | | PHH3 is bounded by 13.5 | | total |
| --- | --- | --- | --- | --- | --- |
|  |  |  | 13 or less | Greater than  or equal to  14 |  |
| Depth of infiltration | No infiltration | Counting  Percentage of depth of infiltration  Percentage of PHH3  bounded by 13.5 | 48 | 17 | 65 |
|  |  |  | 73.8% | 26.2% | 100.0% |
|  |  |  | 90.6% | 35.4% | 64.4% |
|  | | |  |  |  |
| Percentage of total   \| Infiltrating  lamina  propria \| Counting \| \| --- \| --- \| \| Percentage of depth of infiltration \| \| Percentage of PHH3  bounded by 13.5 \| \| Percentage of total \| \| Infiltrating muscle layer \| Counting \| \| Percentage of depth of infiltration \| \| Percentage of PHH3  bounded by 13.5 \| \| Percentage of total \| | | | 47. 5% | 16.8% | 64.4% |
|  |  |  | 4 | 22 | 26 |
|  |  |  | 15.4% | 84.6% | 100.0% |
|  |  |  | 7. 5% | 45.8% | 25.7% |
|  |  |  | 4.0% | 21.8% | 25.7% |
|  |  |  | 1 | 4 | 5 |
|  |  |  | 20.0% | 80.0% | 100.0% |
|  |  |  | 1.9% | 8.3% | 5.0% |
|  |  |  | 1.0% | 4.0% | 5.0% |
|  |  |  | 0 | 5 | 5 |
| Full layer | | Counting   \| Percentage of depth of infiltration \| \| --- \| \| The percentage of PHH3 bounded by 13.5 \| |  |  |  |
|  |  |  | 0.0% | 100.0% | 100.0% |
|  |  |  | 0.0% | 10.4% | 5.0% |
|  |  |  | 0.0% | 5.0% | 5.0% |
| Percentage of total | | |  |  |  |
| Total | Counting   \| Percentage of infiltration depth \| \| --- \| \| Percentage of PHH3  bounded by 13.5 \|   Percentage of total | | 53 | 48 | 101 |
|  |  |  | 52. 5% | 47. 5% | 100.0% |
|  |  |  | 100.0% | 100.0% | 100.0% |
|  |  |  | 52. 5% | 47. 5% | 100.0% |

|  | value | Degrees  of  freedom | Progressive  significance  (bilateral) | Precise  significance  (bilateral) | Exact  significance  (unilateral) | Point  probabilit  y |
| --- | --- | --- | --- | --- | --- | --- |
| Pearson chi- square Likelihood ratio Fisher precision test  Linear correlation Number of valid cases | 33.882a  37.733  b  .  26.230c  101 | 3  3  1 | 000. 000.  000. | b  .  b  .  b  .  000. | 000. | 000. |

a. 4 cells (50.0%) have an expected count of less than 5. The minimum expected count is 2.38.

b. The calculation cannot be performed because the temporary file cannot be opened.

c. Standardized statistics are 5.121.

**Symmetrical** **measurement**

|  | | value | Progressive significance | Precise significance |
| --- | --- | --- | --- | --- |
| Nominal to Phi | | 579. | 000. | c  . |
| nominal | Clem V |  |  |  |
|  |  | 579. | 000. | c  . |
|  |  | 501.  101 | 000. | c  . |
| Number of column connectio ns  Number of valid cases | |  |  |  |

c. Calculations cannot be performed because the temporary file

cannot be opened.

**Infiltrating** **depth** ***** **HE** **is** **bounded** **by** **14.5**

**crosstabs**

|  | | | HE is bounded by 14.5 | | total |
| --- | --- | --- | --- | --- | --- |
|  |  |  | 14 or less | 15 or  more |  |
| Depth of infiltration | No infiltration | Counting   \| Percentage of depth of infiltration \| \| --- \| \| Percentage of HE bounded by 14.5 \|   Percentage of total | 51 | 14 | 65 |
|  |  |  | 78. 5% | 21. 5% | 100.0% |
|  |  |  | 91. 1% | 31. 1% | 64.4% |
|  |  |  | 50. 5% | 13.9% | 64.4% |

| Infiltrating Counting | | 4 | 22 | 26 |
| --- | --- | --- | --- | --- |
|  | |  |  |  |
| lamina propria | Percentage of depth of infiltration | 15.4% | 84.6% | 100.0% |
| Percentage of HE bounded by 14.5 | | 7. 1% | 48.9% | 25.7% |
| Percentage of total   \| Infiltrating muscle layer \| Counting \| \| --- \| --- \| \| Percentage of depth of infiltration \| \| Percentage of HE bounded by 14.5 \| \| Percentage of total \| | | 4.0% | 21.8% | 25.7% |
|  |  | 1 | 4 | 5 |
|  |  | 20.0% | 80.0% | 100.0% |
|  |  | 1.8% | 8.9% | 5.0% |
|  |  | 1.0% | 4.0% | 5.0% |
|  |  | 0 | 5 | 5 |
| Full layer | Counting   \| Percentage of depth of infiltration \| \| --- \| \| Accounts for the percentage of HE bounded by 14.5 \| |  |  |  |
|  |  | 0.0% | 100.0% | 100.0% |
|  |  | 0.0% | 11. 1% | 5.0% |
|  |  | 0.0% | 5.0% | 5.0% |
| Percentage of total | |  |  |  |
| Total | Counting   \| Percentage of infiltration depth \| \| --- \| \| Percentage of HE bounded by 14.5 \|   Percentage of total | 56 | 45 | 101 |
|  |  | 55.4% | 44.6% | 100.0% |
|  |  | 100.0% | 100.0% | 100.0% |
|  |  | 55.4% | 44.6% | 100.0% |

**Chi-square** **test**

|  | value | Degrees  of  freedom | Progressive  significance  (bilateral) | Precise  significance  (bilateral) | Exact  significance  (unilateral) | Point  probabilit  y |
| --- | --- | --- | --- | --- | --- | --- |
| Pearson chi- square Likelihood ratio Fisher precision test  Linear correlation Number of valid cases | 39.595a  43.756  b  .  30.485c  101 | 3  3  1 | 000. 000.  000. | b  .  b  .  b  .  000. | 000. | 000. |

a. 4 cells (50.0%) have an expected count of less than 5. The minimum expected count is 2.23.

b. The calculation cannot be performed because the temporary file cannot be opened.

c. Standardized statistics are 5.521.

**Symmetrical** **measurement**

|  | | value | Progressive significance | Precise significance |
| --- | --- | --- | --- | --- |
| Nominal to Phi | | 626. | 000. | c  . |
| nominal | Clem V |  |  |  |
|  |  | 626. | 000. | c  . |
|  |  | 531.  101 | 000. | c  . |
| Number of column connectio ns  Number of valid cases | |  |  |  |

c. Calculations cannot be performed because the temporary file

cannot be opened.

**Infiltration** **depth** ***** **KI67** **grouped** **in** **9.5** **percent**

**crosstabs**

|  | | | KI67 is grouped by 9.5 percent | | total |
| --- | --- | --- | --- | --- | --- |
|  |  |  | 9 or less | 10 or  higher |  |
| Depth of infiltration | No infiltration | Counting  Percentage of depth of infiltration  Percentage of KI67 grouped by 9.5 percent | 41 | 24 | 65 |
|  |  |  | 63. 1% | 36.9% | 100.0% |
|  |  |  | 95.3% | 41.4% | 64.4% |

| Percentage of total   \| Infiltrating  lamina  propria \| Counting \| \| --- \| --- \| \| Percentage of depth of infiltration \| \| Percentage of KI67 grouped by 9.5 percent \| \| Percentage of total \| \| Infiltrating muscle layer \| Counting \| \| Percentage of depth of infiltration \| \| Percentage of KI67 grouped by 9.5 percent \| \| Percentage of total \| | | 40.6% | 23.8% | 64.4% |
| --- | --- | --- | --- | --- | --- | --- | --- | --- | --- | --- | --- | --- | --- | --- |
|  |  | 2 | 24 | 26 |
|  |  | 7.7% | 92.3% | 100.0% |
|  |  | 4.7% | 41.4% | 25.7% |
|  |  | 2.0% | 23.8% | 25.7% |
|  |  | 0 | 5 | 5 |
|  |  | 0.0% | 100.0% | 100.0% |
|  |  | 0.0% | 8.6% | 5.0% |
|  |  | 0.0% | 5.0% | 5.0% |
|  |  | 0 | 5 | 5 |
| Full layer | Counting   \| Percentage of depth of infiltration \| \| --- \| \| Accounts for 9.5 percent of KI67 groups \|   Percentage of total |  |  |  |
|  |  | 0.0% | 100.0% | 100.0% |
|  |  | 0.0% | 8.6% | 5.0% |
|  |  | 0.0% | 5.0% | 5.0% |
| Total | Counting   \| Percentage of infiltration depth \| \| --- \| \| Percentage of KI67 grouped by 9.5 percent \|   Percentage of total | 43 | 58 | 101 |
|  |  | 42.6% | 57.4% | 100.0% |
|  |  | 100.0% | 100.0% | 100.0% |
|  |  | 42.6% | 57.4% | 100.0% |

**Chi-square** **test**

|  | value | Degrees  of  freedom | Progressive  significance  (bilateral) | Precise  significance  (bilateral) | Exact  significance  (unilateral) | Point  probabilit  y |
| --- | --- | --- | --- | --- | --- | --- |
| Pearson chi- square Likelihood ratio Fisher precision test  Linear correlation Number of valid cases | 31.529a  38.067  b  .  24.119c  101 | 3  3  1 | 000. 000.  000. | b  .  b  .  b  .  000. | 000. | 000. |

a. 4 cells (50.0%) have an expected count of less than 5. The minimum expected count is 2.13.

b. The calculation cannot be performed because the temporary file cannot be opened.

c. Standardized statistics are 4.911.

**Symmetrical** **measurement**

|  | | value | Progressive significance | Precise significance |
| --- | --- | --- | --- | --- |
| Nominal to Phi | | 559. | 000. | c  . |
| nominal | Clem V |  |  |  |
|  |  | 559. | 000. | c  . |
|  |  | 488.  101 | 000. | c  . |
| Number of column connectio ns  Number of valid cases | |  |  |  |

c. Calculations cannot be performed because the temporary file

cannot be opened.

**Infiltrating** **depth** ***** **P53** **is** **bounded** **by** **50** **percent**

**crosstabs**

|  | | | P53 is bounded by 50 percent | | total |
| --- | --- | --- | --- | --- | --- |
|  |  |  | < 50% | 50% or  higher |  |
| Depth of infiltration | No infiltration | Counting  Percentage of depth of infiltration  Percentage of P53 bounded by 50 percent | 60 | 5 | 65 |
|  |  |  | 92.3% | 7.7% | 100.0% |
|  |  |  | 75.9% | 22.7% | 64.4% |

| Percentage of total   \| Infiltrating  lamina  propria \| Counting \| \| --- \| --- \| \| Percentage of depth of infiltration \| \| Percentage of P53 bounded by 50 percent \| \| Percentage of total \| \| Infiltrating muscle layer \| Counting \| \| Percentage of depth of infiltration \| \| Percentage of P53 bounded by 50 percent \| \| Percentage of total \| | | 59.4% | 5.0% | 64.4% |
| --- | --- | --- | --- | --- | --- | --- | --- | --- | --- | --- | --- | --- | --- | --- |
|  |  | 12 | 14 | 26 |
|  |  | 46.2% | 53.8% | 100.0% |
|  |  | 15.2% | 63.6% | 25.7% |
|  |  | 11.9% | 13.9% | 25.7% |
|  |  | 3 | 2 | 5 |
|  |  | 60.0% | 40.0% | 100.0% |
|  |  | 3.8% | 9. 1% | 5.0% |
|  |  | 3.0% | 2.0% | 5.0% |
|  |  | 4 | 1 | 5 |
| Full layer | Counting   \| Percentage of depth of infiltration \| \| --- \| \| Accounts for the percentage of P53 bounded by 50 percent \|   Percentage of total |  |  |  |
|  |  | 80.0% | 20.0% | 100.0% |
|  |  | 5. 1% | 4. 5% | 5.0% |
|  |  | 4.0% | 1.0% | 5.0% |
| Total | Counting   \| Percentage of infiltration depth \| \| --- \| \| Percentage of P53 bounded by 50 percent \|   Percentage of total | 79 | 22 | 101 |
|  |  | 78.2% | 21.8% | 100.0% |
|  |  | 100.0% | 100.0% | 100.0% |
|  |  | 78.2% | 21.8% | 100.0% |

**Chi-square** **test**

|  | value | Degrees  of  freedom | Progressive  significance  (bilateral) | Precise  significance  (bilateral) | Exact  significance  (unilateral) | Point  probabilit  y |
| --- | --- | --- | --- | --- | --- | --- |
| Pearson chi- square Likelihood ratio Fisher precision test  Linear  correlation | 24.246a  22.997  b  .  8.714c | 3  3  1 | 000. 000.  003. | b  .  b  .  b  .  004. | 004. | 002. |

a. 4 cells (50.0%) have an expected count of less than 5. The minimum expected count is 1.09.

b. The calculation cannot be performed because the temporary file cannot be opened.

c. Standardized statistics are 2.952.

**Symmetrical** **measurement**

|  | | value | Progressive significance | Precise significance |
| --- | --- | --- | --- | --- |
| Nominal to Phi | | 490. | 000. | c  . |
| nominal | Clem V |  |  |  |
|  |  | 490. | 000. | c  . |
|  |  | 440.  101 | 000. | c  . |
| Number of column connectio ns  Number of valid cases | |  |  |  |

c. Calculations cannot be performed because the temporary file

cannot be opened.

**Clinical** **staging** ***** **PHH3** **is** **bounded** **by** **13.5**

**Crosstabs**

|  | PHH3 is bounded by 13.5 | | total |
| --- | --- | --- | --- |
|  | 13 or less | Greater than  or equal to  14 |  |
| Clinical No Counting  staging evaluation Percentage of clinical stage  Percentage of PHH3 bounded by 13.5  A percentage of the total | 32 | 0 | 32 |
|  | 100.0% | 0.0% | 100.0% |
|  | 60.4% | 0.0% | 31.7% |
|  | 31.7% | 0.0% | 31.7% |

| Ta+T1 Count  Percentage of clinical stages  Percentage of PHH3 bounded by 13.5  Percentage of total  T2+T3+T4 Count   \| Percentage of clinical stage \| \| --- \| \| Percentage of PHH3  bounded by 13.5 \|   Percentage of total | | 20 | 39 | 59 |
| --- | --- | --- | --- | --- | --- | --- |
|  |  | 33.9% | 66. 1% | 100.0% |
|  |  | 37.7% | 81.3% | 58.4% |
|  |  | 19.8% | 38.6% | 58.4% |
|  |  | 1 | 9 | 10 |
|  |  | 10.0% | 90.0% | 100.0% |
|  |  | 1.9% | 18.8% | 9.9% |
|  |  | 1.0% | 8.9% | 9.9% |
| Total | Counting   \| Percentage of clinical  stages \| \| --- \| \| Percentage of PHH3  bounded by 13.5 \|   Percentage of total | 53 | 48 | 101 |
|  |  | 52. 5% | 47. 5% | 100.0% |
|  |  | 100.0% | 100.0% | 100.0% |
|  |  | 52. 5% | 47. 5% | 100.0% |

**Chi-square** **test**

|  | value | Degrees  of  freedom | Progressive  significance  (bilateral) | Precise  significance  (bilateral) | Exact  significance  (unilateral) | Point  probabilit  y |
| --- | --- | --- | --- | --- | --- | --- |
| Pearson chi- square Likelihood ratio Fisher precision test  Linear correlation Number of valid cases | 44.380a  57.704  b  .  40.388c  101 | 2  2  1 | 000. 000.  000. | b  .  b  .  b  .  000. | 000. | 000. |

a. 1 cell (16.7%) has an expected count of less than 5. The minimum expected count is 4.75.

b. The calculation cannot be performed because the temporary file cannot be opened.

c. The standardized statistics are 6.355.

**Symmetrical** **measurement**

|  | | value | Progressive significance | Precise significance |
| --- | --- | --- | --- | --- |
| Nominal to Phi | | 663. | 000. | c  . |
| nominal | Klem V |  |  |  |
|  |  | 663. | 000. | c  . |
|  |  | 553.  101 | 000. | c  . |
| Number of column connectio ns  Number of valid cases | |  |  |  |

c. Calculations cannot be performed because the temporary file

cannot be opened.

**Clinical** **staging** ***** **HE** **is** **bounded** **by** **14.5**

**Crosstabs**

|  | | | HE is bounded by 14.5 | | Total |
| --- | --- | --- | --- | --- | --- |
|  |  |  | 14 or less | 15 or  more |  |
| Clinical  staging | No | Counting | 32 | 0 | 32 |
|  | evaluation Percentage of clinical stage | | 100.0% | 0.0% | 100.0% |
|  | Percentage of HE bounded by 14.5 | | 57. 1% | 0.0% | 31.7% |
|  | Percentage of total   \| Ta+T1 \| Count   \| Percentage of clinical  stages \| \| --- \| \| Percentage of HE bounded by 14.5 \|   A percentage of the total \| \| --- \| --- \| --- \| --- \|   T2+T3+T4 Count   \| Percentage of clinical stage \| \| --- \| \| Percentage of HE bounded by 14.5 \|   Percentage of total | | 31.7% | 0.0% | 31.7% |
|  |  |  | 23 | 36 | 59 |
|  |  |  | 39.0% | 61.0% | 100.0% |
|  |  |  | 41. 1% | 80.0% | 58.4% |
|  |  |  | 22.8% | 35.6% | 58.4% |
|  |  |  | 1 | 9 | 10 |
|  |  |  | 10.0% | 90.0% | 100.0% |
|  |  |  | 1.8% | 20.0% | 9.9% |
|  |  |  | 1.0% | 8.9% | 9.9% |
| Total | Counting   \| Percentage of clinical  stages \| \| --- \| \| Percentage of HE bounded by 14.5 \|   Percentage of total | | 56 | 45 | 101 |
|  |  |  | 55.4% | 44.6% | 100.0% |
|  |  |  | 100.0% | 100.0% | 100.0% |
|  |  |  | 55.4% | 44.6% | 100.0% |

**Chi-square** **test**

|  | value | Degrees  of  freedom | Progressive  significance  (bilateral) | Precise  significance  (bilateral) | Exact  significance  (unilateral) | Point  probabilit  y |
| --- | --- | --- | --- | --- | --- | --- |
| Pearson chi- square Likelihood ratio | 40.547a  53.410 | 2  2 | 000.  000. | b  .  b  . |  |  |

| Fisher precision test  Linear correlation Number of valid cases | b  .  38.080c  101 | 1 | 000. | b  .  000. | 000. | 000. |
| --- | --- | --- | --- | --- | --- | --- |

a. 1 cell (16.7%) has an expected count of less than 5. The minimum expected count is 4.46.

b. The calculation cannot be performed because the temporary file cannot be opened.

c. 6.171 for standardized statistics.

**Symmetrical** **measurement**

|  | | value | Progressive significance | Precise significance |
| --- | --- | --- | --- | --- |
| Nominal to Phi | | 634. | 000. | c  . |
| nominal | Clem V |  |  |  |
|  |  | 634. | 000. | c  . |
|  |  | 535.  101 | 000. | c  . |
| Number of column connectio ns  Number of valid cases | |  |  |  |

c. Calculations cannot be performed because the temporary file

cannot be opened

**Clinical** **staging** ***** **KI67** **grouped** **by** **9.5** **percent**

**crosstabs**

|  | KI67 is grouped by 9.5 percent | | Total |
| --- | --- | --- | --- |
|  | 9 or less | 10 or  higher |  |
| Clinical No Counting  staging evaluation Percentage of clinical stage  Percentage of KI67 grouped by 9.5 percent  Percentage of total | 31 | 1 | 32 |
|  | 96.9% | 3. 1% | 100.0% |
|  | 72. 1% | 1.7% | 31.7% |
|  | 30.7% | 1.0% | 31.7% |

| Ta+T1 Count  Percentage of clinical stages  Percentage of KI67 grouped by 9.5 percent  Percentage of total  T2+T3+T4 Count   \| Percentage of clinical stage \| \| --- \| \| Percentage of KI67 grouped by 9.5 percent \|   A percentage of the total | | 12 | 47 | 59 |
| --- | --- | --- | --- | --- | --- | --- |
|  |  | 20.3% | 79.7% | 100.0% |
|  |  | 27.9% | 81.0% | 58.4% |
|  |  | 11.9% | 46. 5% | 58.4% |
|  |  | 0 | 10 | 10 |
|  |  | 0.0% | 100.0% | 100.0% |
|  |  | 0.0% | 17.2% | 9.9% |
|  |  | 0.0% | 9.9% | 9.9% |
| Total | Counting   \| Percentage of clinical  stages \| \| --- \| \| Percentage of KI67 grouped by 9.5 percent \|   Percentage of total | 43 | 58 | 101 |
|  |  | 42.6% | 57.4% | 100.0% |
|  |  | 100.0% | 100.0% | 100.0% |
|  |  | 42.6% | 57.4% | 100.0% |

**Chi-square** **test**

|  | value | Degrees  of  freedom | Progressive  significance  (bilateral) | Precise  significance  (bilateral) | Exact  significance  (unilateral) | Point  probabilit  y |
| --- | --- | --- | --- | --- | --- | --- |
| Pearson chi- square Likelihood ratio Fisher precision test  Linear correlation Number of valid cases | 57.938a  69.282  b  .  50.939c  101 | 2  2  1 | 000. 000.  000. | b  .  b  .  b  .  000. | 000. | 000. |

a. 1 cell (16.7%) has an expected count of less than 5. The minimum expected count is 4.26.

b. The calculation cannot be performed because the temporary file cannot be opened.

c. Standardized statistics are 7.137.

**Symmetrical** **measurement**

|  | | value | Progressive significance | Precise significance |
| --- | --- | --- | --- | --- |
| Nominal to Phi | | 757. | 000. | c  . |
| nominal | Clem V |  |  |  |
|  |  | 757. | 000. | c  . |
|  |  | 604.  101 | 000. | c  . |
| Number of column connectio ns  Number of valid cases | |  |  |  |

c. Calculations cannot be performed because the temporary file

cannot be opened.

**Clinical** **staging** ***** **P53** **is** **bounded** **by** **50** **percent**

**Crosstabs**

|  | | | P53 is bounded by 50 percent | | Total |
| --- | --- | --- | --- | --- | --- |
|  |  |  | < 50% | 50% or  higher |  |
| Clinical  staging | No | Counting | 31 | 1 | 32 |
|  | evaluation Percentage of clinical stage | | 96.9% | 3. 1% | 100.0% |
|  | Percentage of P53 bounded by 50 percent | | 39.2% | 4. 5% | 31.7% |
|  | Percentage of total   \| Ta+T1 \| Count   \| Percentage of clinical  stages \| \| --- \| \| Percentage of P53 bounded by 50 percent \|   Percentage of total \| \| --- \| --- \| --- \| --- \|   T2+T3+T4 Count   \| Percentage of clinical stage \| \| --- \| \| Percentage of P53 bounded by 50 percent \|   Percentage of total | | 30.7% | 1.0% | 31.7% |
|  |  |  | 41 | 18 | 59 |
|  |  |  | 69. 5% | 30. 5% | 100.0% |
|  |  |  | 51.9% | 81.8% | 58.4% |
|  |  |  | 40.6% | 17.8% | 58.4% |
|  |  |  | 7 | 3 | 10 |
|  |  |  | 70.0% | 30.0% | 100.0% |
|  |  |  | 8.9% | 13.6% | 9.9% |
|  |  |  | 6.9% | 3.0% | 9.9% |
| Total | Counting   \| Percentage of clinical  stages \| \| --- \| \| Percentage of P53 bounded by 50 percent \|   A percentage of the total | | 79 | 22 | 101 |
|  |  |  | 78.2% | 21.8% | 100.0% |
|  |  |  | 100.0% | 100.0% | 100.0% |
|  |  |  | 78.2% | 21.8% | 100.0% |

**Chi-square** **test**

|  | value | Degrees  of  freedom | Progressive  significance  (bilateral) | Precise  significance  (bilateral) | Exact  significance  (unilateral) | Point  probabilit  y |
| --- | --- | --- | --- | --- | --- | --- |
| Pearson chi- square Likelihood ratio | 9.571a  12.175 | 2  2 | 008.  002. | b  .  b  . |  |  |

| Fisher precision test  Linear correlation Number of valid cases | b  .  7.205c  101 | 1 | 007. | b  .  009. | 006. | 004. |
| --- | --- | --- | --- | --- | --- | --- |

a. 1 cell (16.7%) has an expected count of less than 5. The minimum expected count is 2.18.

b. The calculation cannot be performed because the temporary file cannot be opened.

c. Standardized statistics are 2.684.

**Symmetrical** **measurement**

|  | | value | Progressive significance | Precise significance |
| --- | --- | --- | --- | --- |
| Nominal to Phi | | 308. | 008. | c  . |
| nominal | Clem V |  |  |  |
|  |  | 308. | 008. | c  . |
|  |  | 294.  101 | 008. | c  . |
| Number of column connectio ns  Number of valid cases | |  |  |  |

c. Calculations cannot be performed because the temporary file

cannot be opene
